# Exploring the functional nature of synaesthetic colour: Dissociations from colour perception and imagery

**DOI:** 10.1016/j.cognition.2018.03.022

**Published:** 2018-08

**Authors:** Rocco Chiou, Anina N. Rich, Sebastian Rogers, Joel Pearson

**Affiliations:** a The Neuroscience and Aphasia Research Unit (NARU), Division of Neuroscience and Experimental Psychology, University of Manchester, UK; b Perception in Action Research Centre & Department of Cognitive Science, Macquarie University, Sydney, NSW, Australia; c ARC Centre of Excellence in Cognition and its Disorders, Australia; d School of Psychology, University of New South Wales, Sydney, NSW, Australia

**Keywords:** Synaesthesia, Colour, Binocular rivalry, Priming, Attention, Consciousness

## Abstract

Individuals with grapheme-colour synaesthesia experience anomalous colours when reading achromatic text. These unusual experiences have been said to resemble ‘normal’ colour perception or colour imagery, but studying the nature of synaesthesia remains difficult. In the present study, we report novel evidence that synaesthetic colour impacts conscious vision in a way that is different from both colour perception and imagery. Presenting ‘normal’ colour prior to binocular rivalry induces a location-dependent suppressive bias reflecting local habituation. By contrast, a grapheme that evokes synaesthetic colour induces a facilitatory bias reflecting priming that is not constrained to the inducing grapheme’s location. This priming does not occur in non-synaesthetes and does not result from response bias. It is sensitive to diversion of visual attention away from the grapheme, but resistant to sensory perturbation, reflecting a reliance on cognitive rather than sensory mechanisms. Whereas colour imagery in non-synaesthetes causes local priming that relies on the locus of imagined colour, imagery in synaesthetes caused global priming not dependent on the locus of imagery. These data suggest a unique psychophysical profile of high-level colour processing in synaesthetes. Our novel findings and method will be critical to testing theories of synaesthesia and visual awareness.

## 1 Introduction

Studying colour perception epitomises the challenge of understanding the mechanisms that underpin the contents of consciousness – how is the subjective experience of colours created from variations in the wavelengths of light? Grapheme-colour synaesthesia provides a unique window into the mechanisms by which the brain creates colour. People with this unusual condition have involuntary colour experiences triggered by reading achromatic letters and numbers. The relationship of synaesthetic colour with ‘normal’ colour (triggered by relative wavelengths of light) and imagined colour (generated at will) is a topic of much neural and psychophysical research. Attempts to characterise the nature of synaesthetic colour have been faced with the classic difficulties of studying conscious experience. Most of the data come from subjective reports of perceptual judgements that are prone to post-perceptual cognitive strategies, contextual influences, or paradigms that do not have appropriate control conditions, which are confounded by decisional bias. Other ‘proxy’ measures of synaesthesia, such as the synaesthetic congruency/Stroop effect, reflect the involuntary nature of synaesthetic colour rather than its qualia *per se* (for review of relevant evidence, see Chiou and Rich, 2014, Mattingley, 2009).

Although synaesthetes readily differentiate different forms of colour experiences, many describe synaesthetic colour as vivid as ‘normal’ colour (Rich, Bradshaw, & Mattingley, 2005). This apparent resemblance between synaesthetic and actual colour has led to prominent hypotheses that synaesthetic colours involve a key functional cortical area for colour perception – the ventral occipitotemporal V4 (Hubbard et al., 2011, Hubbard and Ramachandran, 2005, Rouw et al., 2011). Some studies claim that synaesthetic colours trigger V4 activation (or *in the vicinity of* V4) in some synaesthetes (e.g., Brang et al., 2010, Dovern et al., 2012, Hubbard et al., 2005, Tomson et al., 2013, Van Leeuwen et al., 2011, van Praag et al., 2016), while others have failed to find similar effects (e.g., Hupé et al., 2012, Rich et al., 2006). In light of the discrepancy, Rouw et al. (2011) conducted a review highlighting the variability between studies with fewer than half showing V4 activation in synaesthetes. Similarly, in a critical review, Hupé and Dojat (2015) challenged all V4 activation findings in the synaesthesia literature on the grounds of statistical power and liberal threshold, concluding that there is *no* evidence whatsoever for the involvement of V4. Thus, despite the ubiquity of claims that synaesthesia induces V4 activity, there are significant concerns regarding the robustness of evidence, creating doubt about whether synaesthesia does actually depend on the same areas as ‘normal’ colour perception.

In fact, behavioural research has demonstrated marked differences between synaesthetic and ‘normal’ colour. For example, ‘normal’ colour captures visual attention when it is the singly distinctive feature, leading to efficient visual search (pre-attentive pop-out: Treisman & Gelade, 1980). Although synaesthetic colour has been seen to bestow advantage for some synaesthetes in visual search (Laeng et al., 2004, Laeng, 2009, Palmeri et al., 2002, Ramachandran and Hubbard, 2001), such advantage is not always replicated (Edquist, Rich, Brinkman, & Mattingley, 2006) and seems to reflect higher-level strategies (e.g., grouping) rather than pop-out (Rich and Karstoft, 2013, Ward et al., 2010). Additionally, perceiving ‘normal’ colour is affected by its surroundings (chromatic contrast: Hurlbert & Wolf, 2004), but such contrast phenomena do not influence synaesthetic colour (Erskine et al., 2012, Nijboer et al., 2011). Finally, Arnold, Wegener, Brown, and Mattingley (2012) found that adjusting a colour patch to match synaesthetic colour (induced by either visually or aurally presented letter or number) and recalled colour (perceptual memory of a colour seen earlier) were all less precise than matching to a ‘normal’ colour currently in view. These data suggest that synaesthesia is closer to *recollecting* colour than to *perceiving* colour, at least in precision.

Interestingly, other studies looking at individual data have suggested that synaesthetic colours interact with ‘normal’ colours to influence perception. Most relevant to the current study, Kim, Blake, and Palmeri (2006) tested two synaesthetes using a binocular rivalry task that tested whether synaesthetic colour might enable grouping of separate elements into a unified global percept, similar to ‘normal’ colour stimuli that form a conjoint entity and prolong perceptual predominance. While undergoing binocular rivalry, their two synaesthetes viewed graphemic stimuli and reported their dominant percept. The authors found that both synaesthetic and ‘normal’ colour seemed to increase the amount of perceptual grouping and concluded that synaesthetic colours can behave like ‘normal’ colours. In the present study, we build on this intriguing finding with a novel rigorous approach that allows us to explore the nature of the synaesthetic colour and compare it to the effects of ‘normal’ and voluntarily imagined colour.

We present evidence that synaesthetic colour impacts conscious vision in a manner unlike perceiving ‘normal’ colour and with intriguing differences from non-synaesthetes performing voluntary colour imagery. We devised a paradigm to assess the influences of synaesthetic colour on vision during binocular rivalry. Our novel method allowed us to gauge whether the impact is facilitatory or suppressive, whether the effect occurs locally at the inducer (letter) location or spreads globally to other parts of the visual field, and whether it differs from perception and imagery. Specifically, we know that ‘normal’ colour experiences are strongly constrained to the patch of colour reflecting those wavelengths of light. Thus, we anticipate its effect on binocular rivalry should occur within the area where the ‘normal’ colour stimulus is located. However, it is unclear whether synaesthetic colour would be analogously confined to the location at which the inducing grapheme is located or whether it would show a ‘spillover’ effect to other locations. Thus, there may be crucial differences in terms of their reliance on location. In Experiment 1, we find that synaesthetic and normal colours have qualitatively different influences on subsequent conscious perception. In Experiment 2, we replicate our synaesthetic effect, and additionally show that controls do not show the same effects from achromatic graphemes, demonstrating it is specific to synaesthetes. In Experiment 3, we find that, unlike voluntary colour imagery, the synaesthetic effect on subsequent binocular rivalry is not disrupted by sensory luminance-level perturbation. In Experiments 4A and 4B, we find that attenuating synaesthesia through high-level cognitive interference during the elicitation of synaesthetic colour reduces its effect on subsequent binocular rivalry, consistent with previous reports about the importance of attention for evoking synaesthesia (e.g., Rich & Mattingley, 2010). Finally, in Experiment 5, we find that synaesthetes show qualitatively different effects when doing a voluntary colour imagery version of the binocular rivalry task, relative to controls performing the same task and from previous reports of voluntary colour imagery. This may reflect anomalous imagery in the synaesthesia group, or the combination of normal voluntary imagery with additional synaesthetic experiences.

## 2 General method

### 2.1 Participants

We tested 14 participants with grapheme-colour synaesthesia (mean age ± SD: 32 ± 14 years, 11 females, 12 native speakers of English, 2 native speakers of Mandarin; Experiment 1, *n* = 6; Experiment 2, *n* = 10; Experiment 3, *n* = 8; Experiments 4A & 4B, *n* = 10; Experiment 5, *n* = 6). Some synaesthetes participated in more than one experiment, summarised in Table S1 of the online supplemental information (SI); also see SI for discussion regarding the categorisation of synaesthetes into subgroups based on subjective descriptions. The number of participants varies somewhat across the experiments due to availability at the time of recruitment. Previous imagery studies using the same techniques as ours ranged in sample size between 5 and 20 participants (e.g., Chang et al., 2013, Pearson et al., 2008, Pearson et al., 2011, Sherwood and Pearson, 2010), suggesting our sample sizes, albeit small, give a reasonable chance of detecting effects. All synaesthetes completed a standard questionnaire, used in previous studies (Mattingley et al., 2001, Rich and Karstoft, 2013, Rich et al., 2005), that covers personal and demographic details and experiences of synaesthesia. We also tested non-synaesthetic controls in Experiments 2 and 5. In Experiment 2, we compared 10 controls with 10 synaesthetes; the two groups were matched on sex, age, and native language (controls’ age: 31 ± 12 years old, 8 females, 8/2 native speakers of English/Mandarin). In Experiment 5, we matched controls to 6 synaesthetes using two different sets of criteria. In our demographic-match group, 12 controls (2 for each of the 6 synaesthetes) were matched on demographic details (controls’ age: 34 ± 4 years, 10 females, all native speakers of English). In our imagery-match group (non-overlapping with the demographic controls), we selected 12 controls from a larger sample of 30 participants based on the magnitude of their imagery priming in one specific condition of the voluntary mental imagery experiment (for details, see the Methods of Experiment 5). All participants were naïve to the purpose of the study and reported having normal or corrected-to-normal visual acuity and colour perception. All synaesthetes were recruited via the database of Synaethesia Participant Register of Macquarie University; all controls were recruited from the community/network of the University of New South Wales. We checked with all controls that they had no synaesthetic experiences. All gave informed consent before participating and received payment for their participation. The study was approved by the local advisory panel for human research ethics.

### 2.2 Synaesthetic colour matching and assessment of consistency

All synaesthetes completed a grapheme-colour matching task to reveal their idiosyncratic grapheme-colour associations and were retested at a later point, allowing us to gauge the consistency of their associations across time. For this task, they were presented with 26 letters of the English alphabet and Arabic digits (0–9) and asked to choose a colour they felt best matched the synaesthetic colours for each grapheme using a computerised colour palette. They moved a cursor on a colour gradient to fine-tune the colour until they were satisfied with the selected colour. This colour matching task was administered in separate sessions at least two months apart. Synaesthetes were highly consistent in the colour hue selected between sessions (M = 94%; SD = 6), based on categorical coding as reported in our previous papers (e.g., Mattingley et al., 2001, Rich and Karstoft, 2013, Rich et al., 2005). This replicates the well-established effect that synaesthetic colour is consistent across repeated measurements (Mattingley et al., 2001, Rich et al., 2005); it also confirmed the basis for the main experiments, which rely on consistent experiences. Using each individual’s range of available synaesthetic colours, we selected two suitable graphemes that reliably induced distinct opponent colours. Based on numerous prior binocular rivalry studies, we know rivalry is more likely to occur when a red/yellow colour is paired with a green/blue colour due to their opponency. Therefore, when selecting colours from each individual’s set of synaesthetic colours we picked colours that appeared as the primary colours of red and green (or yellow and blue, depending on suitability). We avoided using non-primary colours situated in the intermediate range (e.g., orange, halfway between red and yellow). Note that we did not numerically compute opponency, but instead selected the best estimates from each participant’s set of synaesthetic colours. This is due to the specific need of the present study that matching the displayed colours to subjective synaesthetic experiences is more important than matching by numerically-defined colour opponency.

### 2.3 Analysis

We present the standard frequentist statistics, including 95% confidence intervals (CI) around the condition means in graphs, as well as the statistical results as recommended by Cumming (2014) and Cumming and Calin-Jageman (2016). For Experiments 1, 3 and 4, which were paired designs, we use the mean difference (M*_diff_*) and the standard deviation of the mean difference (*s_diff_*). For Experiments 2 and 5, which were designs for independent-groups, we report the margin of error on the difference between the two means (MoE*_diff_*). We use a standardised estimate of effect size, Cohen’s *d* unbiased (*d*), calculated using the average standard deviation for paired samples (Experiment 1, 3, and 4) and the pooled standard deviation as the denominator for independent groups (Experiment 2 and 5) as per the guidelines (Cumming & Calin-Jageman, 2016).

## 3 Experiment 1

In Experiment 1, we capitalised on a key characteristic of the colour vision system: the habituation of physiological response to constant presence of a colour. This results in a suppressive effect on subsequent stimuli of the same colour (i.e., visual adaptation, see Webster, 2011). This habituation for normal colour is restricted to the local region of the visual field (retinotopically-based local adaptation, see Werner, 2003). Hong and Blake (2008) tested four synaesthetes and found that habituating to synaesthetic colour did not alter the sensitivity for ‘habituated vs. opponent’ colours, unlike normal colour, which did. Although this null result might potentially suffer from a lack of statistical power, it hints that synaesthesia and normal colour might rely on separable neural mechanisms, particularly in the early stages of the visual system that contain antagonistic neurons. It is worth noting that the logic behind the design by Hong and Blake (2008), as well as numerous other studies using similar approaches, is to see whether synaesthetic colour performs the same ‘feat’ that actual colour achieves. This approach, as reviewed in the Introduction, has produced equivocal results - synaesthetic colours either give null results or show a subtle effect in the same direction as actual colour (but fall short of the same level of effect size). In the present experiments, we devised a novel paradigm combining colour habituation with subsequent brief binocular rivalry presentations, which allowed us to test for different patterns of synaesthetic versus normal colour within an individual. Our methods allowed us to directly compare whether prolonged exposure (habituation) to different types of colour experience impacts conscious vision in opposite directions (suppressive vs. facilitatory, qualitative difference) or in the same direction, but differing in magnitude (quantitative difference). It also allowed us to explore whether different types of colour experiences are similarly constrained by retinotopic location.

### 3.1 Apparatus & stimuli

We used a chin rest to stabilise head position. Stimuli were generated using MATLAB (R2012b) with the Psychophysics toolbox running on Windows 7 and displayed on a Sony Trinitron G520 CRT monitor with 1280 × 960 resolution and 75-Hz refresh rate. A mirror stereoscope was used for viewing stimuli so that a different pattern could be presented to each eye, resulting in binocular rivalry. The experiment took place in a darkened room, with visual stimuli on the screen being the sole source of luminance. A two-tone bull’s eye fixation circle (diameter: 0.3°; luminance: 9.24 cd/m^2^) at the centre of the screen was shown throughout the events of a trial to facilitate binocular convergence. We carefully adjusted the stereoscope mirrors for each individual so that the patterns from each eye were aligned to generate visual rivalry.

**Fig. 1:**
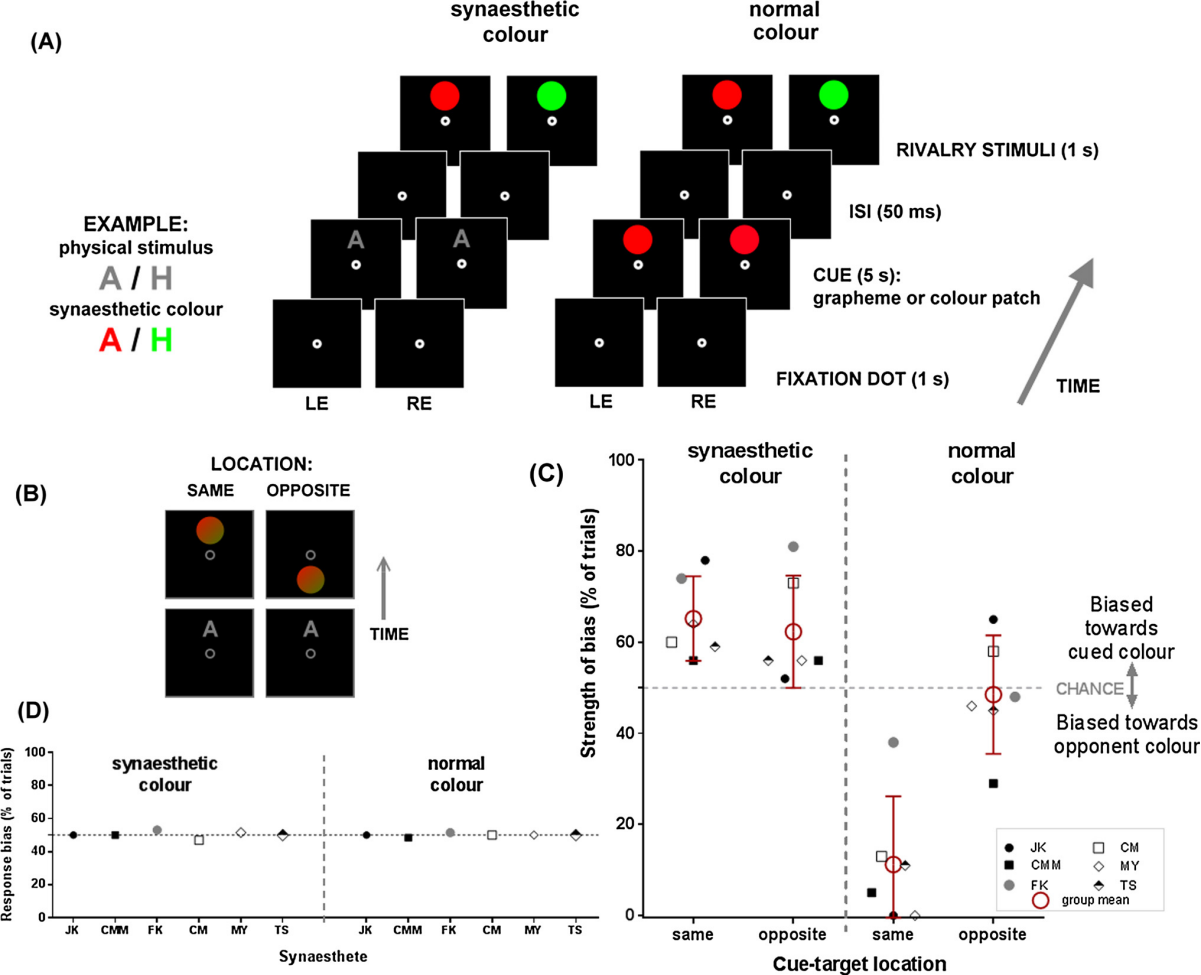
*Experiment 1: the effect of synaesthetic and normal colour on binocular rivalry in synaesthetes.***(A)** Timeline of trial events of the synaesthetic and normal colour conditions. Example stimuli (left) show the physically grey colour of grapheme cues and the synaesthetic colour they elicit for one individual. **(B)** Example displays from the synaesthetic colour block on a catch ‘mock-rivalry’ trial showing the location manipulation, with cue and target in the same location (left) vs. opposite locations (right). The appropriate response for these mock-rivalry trials is ‘mixed’ and provides a measure of response bias towards one or the other colour. **(C)** Data from 6 synaesthetes showing the strength of perceptual bias of each condition, grouped by different types of cues and cue-target location mapping. The dotted chance line (50%) indicates random selection between the two rivalry colours. Values above/below chance indicate a bias in which the dominant colour during binocular rivalry matched the cued/opponent colour. Synaesthetic colour shows a priming effect (bias towards seeing the cued colour) but there is no striking difference between locations. Normal colour shows a robust suppressive habituation effect (bias towards seeing the opponent colour) that is retinotopic (only evident for the same location). Synaesthetes CM, CMM and JK did the synaesthetic colour condition before the normal colour condition, and *vice versa* for synaesthetes FK, MY, and TS. **(D)** Results from the mock-rivalry catch trials. Each data point represents the non-perceptual decisional/response bias for each individual synaesthete, plotted separately for the synaesthetic colour (left) and normal colour conditions (right). In every figure of this paper, LE/RE stands for left/right eye. (For interpretation of the references to colour in this figure legend, the reader is referred to the web version of this article.)

Fig. 1A shows the two rivalry conditions and task details. In the synaesthetic colour condition (Fig. 1A left), synaesthetic participants viewed a mid-grey graphemic cue (the same grapheme displayed to each eye, chosen from the two alphanumeric symbols that induced opponent primary colours). The graphemes subtended diagonally around 2.7° of visual angle and their luminance was 9.24 cd/m^2^. Following the cue was the binocular rivalry stimulus, composed of two coloured circles (diameter: ∼2.7°) presented dichoptically – a different colour to each eye. Rivalry stimuli were coloured using the RGB triplets of the two individually selected opponent synaesthetic colours. To achieve a balance between the two rivalry colours, it was important to equate their strength by adjusting their luminance. Thus, we also calibrated the colours and carefully matched the saliency of each eye’s rivalry stimulus using an eye-dominance test (see below for the calibration procedures), nulling out any potential eye-bias. While the luminance of each rivalry colour was adjusted during the dominance test, they remained similar to the synaesthetic colours of the grapheme cues (see SI for the original RGB values of chosen synaesthetic colours, as well as those of the tweaked colour after dominance test). Note that the synaesthetic colour induced by the cue was always in accordance with one of the two rivalry colours and in opposition to the other. In the normal colour condition (Fig. 1A right), we used the same rivalry displays as the synaesthetic colour condition, but the cues were actual colour patches: participants viewed normal colour cues that consisted of two circles coloured identically in both eyes. The colour, shape, and size of cue stimuli were matched to rivalry targets. Also note that the colours of rivalrous stimuli displayed in the synaesthetic condition were matched to their counterparts (cue and rivalrous disks) in the normal colour conditions in terms of hue, saturation, and luminance values (see below for the calibration and matching protocols).

### 3.2 Procedure & design

The experiment began with a calibration procedure for the synaesthetic colours. The synaesthetic participants viewed a grapheme on a black background and were asked to modify the displayed colours using the MS Office colour palette/gradient until the display colours subjectively matched the induced synaesthetic colour experience to their satisfaction. They were allowed to take their time fine-tuning the colours; the outcomes of colour calibration (i.e., the RGB triplets of each colour) were then used to create the rivalrous target stimuli in the subsequent binocular rivalry experiments.

To measure and nullify any pre-existing bias caused by eye dominance, we administered an eye-dominance test prior to the main binocular rivalry experiments. This test has been extensively used in previous studies to ensure balanced sensory strength of rivalry stimuli (e.g., Chang et al., 2013, Pearson et al., 2008, Pearson et al., 2011, Sherwood and Pearson, 2010; for elaborated discussion about the significance and necessity of this procedure, see Pearson, 2014). The procedure involved adjusting the luminance of the two colours in small steps to determine the point at which perceptual competition was most balanced and hence most susceptible to any biasing influence. In each eye-dominance trial, we presented two coloured circles, using two sets of RGB values derived from the earlier calibration procedure, in a binocular rivalry display for 1 s. Participants were asked to press designated keys to report which of the two colours appeared dominant during the rivalry presentation. If the two colours appeared equally dominant they had to press another key indicating a mixture. Following each response, the colour reported as dominant was shown to *both* eyes for 4 s. This 4-s presentation served as an intervening stimulus that has been shown to reliably induce visual adaptation to the viewed colour, weakening the adapted colour in the following rivalry presentation. The adapted colour would consequently be less likely to appear perceptually dominant in the next rivalry display, resulting in a reversal of perceptual dominance. If the reversal did not occur (i.e., participant reported the same dominant colour in two consecutive trials), the luminance was automatically adjusted for the two colours accordingly, lowering the luminance of the previous dominant colour and increasing the luminance of the other colour. If the adjustment did not lead to a perceptual switch in the next trial, relative luminance was further adjusted until a switch occurred. There were 30 trials in each eye-dominance block. For each participant, we ensured that a reversal of dominance occurred on 80% to 90% of presentations, which reflected balanced visual competition. If perception during rivalry presentations was deemed not balanced (<80% of perceptual reversals), we administered the same procedure and adjusted the luminance derived from the previous test. All participants achieved balance eye-dominance (i.e., >80% of reversals) within 3 repeats of the test.

The main binocular rivalry experiment consisted of four blocks of 80 trials. The trials in the synaesthetic and normal colour conditions were presented in separate blocks, and their order was counterbalanced across participants: 3 of the 6 subjects completed two graphemic cue blocks followed by two normal colour cue blocks; the remaining 3 completed the inverse order. Fig. 1A shows the trial sequence for synaesthetic (left) and normal colour (right) blocks. Each trial began with a fixation display (1 s), followed by the cue (5 s). An inter-stimulus-interval (ISI) of 50 ms was then followed by the rivalry target (1 s). Participants were asked to report, by pushing a designated button, their dominant colour during the period of dichoptic presentation or a balanced mixture of the two. They had 5 s to make a response, followed by an inter-trial-interval (ITI) of 1.5 s. On each trial, the cue and its following rivalry stimulus could be presented 2.6° of visual angle above or below the fixation circle. As Fig. 1B illustrates, the cue location was either the same as the target location (cue and target both being above or below) or they were in opposite locations (cue above, target below or *vice versa*). There were equal numbers of trials in the conditions of the same and opposite location, randomly interleaved within a block. Before the main experiments, the participants went through two practice blocks of 5 trials each.

To measure decisional or response bias, we included mock-rivalry “catch trials” randomly interleaved throughout each block of real rivalry trials (for precedents, see Chang et al., 2013, Pearson et al., 2008, Pearson et al., 2011). The catch trials contained mock-rivalry stimuli of the same size and shape as the rivalry stimuli that were non-rivalrous (i.e., identical in each eye), made by blending the two rivalry colour patches together to create a watercolour-like mixed pattern, mimicking the typical piecemeal-type appearance often seen when neither of the colours is completely dominant (Fig. 1B and Fig. S1). As catch trials should always be perceived as a fusion of the two colours, the ‘correct’ or veridical response is to report a non-unitary ‘mixed’ percept by pressing the designated button. Any non-mixture response to a mock-rivalry stimulus (e.g., reporting it as red) would suggest a bias at a decisional or response-related level. Each block of 80 trials had 80% rivalry trials (64 trials) in which target stimuli were viewed dichoptically, and 20% catch trials (16 trials) in which we presented mock-rivalry stimuli.

The majority of experiments utilising binocular rivalry involve some degree of subjectivity and decisional processes. Our design combats this issue in three ways: First, by using the eye dominance test to ascertain the visual system is equally susceptible to the two rivalry colours; second, by including catch-trials to ensure the key effect, be it habituation or facilitation, is not solely driven by decisional bias; third, by shortening the duration of rivalry to 1 s (much briefer than typical rivalry studies), which reduces the likelihood of a piecemeal or dynamic percept. Thus, our design contained multiple procedures to reduce the effect of decisional bias and possible piecemeal trials. To pre-empt the results of these control measures, we found that our participants rarely reported ‘mixed’ piecemeal percepts on true rivalry trials (balanced dominance), nor ‘biased’ percepts on catch trials (veridical reporting of catch trials, suggesting no response bias). We present the catch trial data for each individual for each experiment (due to the low number of trials, these need to be presented across conditions to ensure a minimum of 32 catch trials contributing to each mean). We used a criterion of >10% from the non-biased level of 50% as our definition of response bias on catch trials (where a participant approaches our 10% deviation criterion, we include a footnote noting the effect of this participant on the main results of the analyses).

### 3.3 Results

Using the same method as previous studies (e.g., Chang et al., 2013, Pearson et al., 2008, Pearson et al., 2011, Sherwood and Pearson, 2010), we coded participants’ reports of dominant colour using the following: responses matching the ‘cued colour’ were coded as 1, responses opposite to the cued colour (i.e., reporting the non-cued colour) were coded as 0, and responses of mixture of two colours without any colour being dominant were coded as 0.5. If a participant randomly selected between two buttons or kept pressing the same button, the average would be around 0.5, indicating chance-level. This provided a baseline for us to examine whether the rivalrous colours significantly deviated towards the cued/non-cued colour, relative to chance, or perceived as an equal blend of the two constituent colours. Before we conducted any analysis, we checked whether a participant experienced a clear percept in most of the rivalry (non-mock) trials. Reports of a mixed percept (i.e., responses coded as 0.5) in the rivalry trials occurred very rarely: 3% in the synaesthetic colour blocks and 2% in the normal colour blocks.

Fig. 1C shows the binocular rivalry bias induced by synaesthetic (left) and normal colours (right) for each individual and the group mean. We first tested for bias in each of the key conditions to verify if we replicated typical location-specific suppressive effects of normal colour, and to see whether synaesthetic colour induced any bias. Perceiving a normal colour cue led to a bias towards seeing *opponent* colour during the subsequent rivalry display when cue and target appeared in the same location (relative to chance, *t*_(5)_ = −6.69, *p* = .001, *M_diff_* = −38.83, CI [−53.75, −23.91], *d_unbiased_* = 2.30, negative indicating a bias away from the cued colour), but this was not the case when they were in opposite locations (relative to chance, *t*_(5)_ < 1, *M_diff_* = −1.50, CI [−14.45, 11.45], *d_unbiased_* = 0.10), replicating prior findings of local, retinotopically-constrained chromatic habituation (Brascamp et al., 2007, Pearson et al., 2008). In contrast, synaesthetic colour cues had a facilitative effect: perceiving a synaesthetic colour cue led to a bias towards seeing the *matching* colour in subsequent rivalry stimulus when cue and target were in the same location (relative to chance, *t*_(5)_ = 4.19, *p =* .009, *M_diff_* = 15.17, CI [5.86, 24.47], *d_unbiased_* = 1.44) and this also seemed likely to occur when they were in opposite locations, albeit with less certainty (*t*_(5)_ = 2.57, *p =* .05, *M_diff_* = 12.33, CI [0.01, 24.66], *d_unbiased_* = 0.88).

A repeated-measures ANOVA on the bias scores with the factors of cue colour type (normal, synaesthetic) and cue-target location (same, opposite) confirmed the pattern evident in Fig. 1C: the location interacts with the effects of each colour cue differently (*F*_(1,5)_ = 10.77, *p* = .02, *M_diff_* [synaesthetic colour_(opposite-same)_ − normal colour_(opposite-same)_] = −40.20%, *s_diff_* = 29.67, CI [9.19, 71.47], *d_unbiased_* = −2.07). The suppression from normal colour was greater when the cue and rivalry stimuli were shown sequentially at the same location, compared to at different locations (location-dependent habituation; *t*_(5) =_ 4.77, *p* = .005, *M_diff_* = 37.33, CI [57.44, 17.23], *d_unbiased_* = 2.36). By contrast, for synaesthetic colour, the facilitatory bias in ‘same versus opposite’ locations did not reach significance (*t*_(5)_ < 1, *M_diff_* = 2.83; CI [−11.39, 17.06], *d_unbiased_* = 0.23). The individual data in Fig. 1C show the variability in both habituation and priming. For normal colour, all synaesthetes show the strong expected bias towards the opponent colour (evidence of habituation) in the same location relative to the opposite location (Fig. 1C right). For synaesthetic colour (Fig. 1C left), although all are above the chance line (towards the cued colour) in both locations, two synaesthetes numerically have greater priming for the opposite versus same locations (FK, CM), two synaesthetes show greater priming for same versus opposite locations (JK, MY), and two synaesthetes show only tiny differences between locations (CMM, TS). At a group level, normal colour consistently drove suppressive bias whereas synaesthetic colour elicited facilitatory bias. In the Discussion section, we expound on the potential causes of the *qualitatively* distinct pattern between synaesthetic and normal colours.

We also examined whether participants showed criterion/decisional bias in favour of the primed colour in catch trials, in which the mock stimuli contained balanced portions of the two component colours. Fig. 1D shows the average for each participant on catch trials, showing they correctly reported the catch trials as mixed blends for both the normal and synaesthetic colour conditions. A bias in the catch-trials would be indicative of a shift of response criteria; hence, the data suggest this is unlikely to be the main driving force of priming for these participants.

## 4 Experiment 2

To replicate our initial finding and to ensure the synaesthetic priming binocular rivalry effect is specific to synaesthetes, we repeated Experiment 1 with a larger synaesthete group (*n* = 10) and an age-, gender-, and native-language-matched control group *without* synaesthesia. In addition, we shortened the duration of the graphemic cues to 0.25 s to ensure that the priming effect was not driven by our synaesthetes voluntarily imagining colour during the cue presentation. Because the aim of Experiment 2 is to replicate the synaesthetic priming effect, we did not include the normal colour cues. Previous work has demonstrated that multiple seconds of actively engaging in mental imagery are necessary to overtly bias subsequent rivalry, reflecting the slow build-up of mental imagery (Pearson et al., 2008).

### 4.1 Design

The design was identical to Experiment 1, with differences that the cue duration was 0.25 s and the normal colour blocks were not included. We tested 10 synaesthetes (4 of whom had participated in Experiment 1) and 10 controls, and included only the graphemic cue (synaesthetic colour) condition. Summed across the two blocks, there were 128 rivalry trials (80%) and 32 catch trials (20%). The two groups went through the same testing procedure for the main experiments (i.e., the eye-dominant tests followed by the binocular rivalry experiment), but only synaesthetes performed the calibration test of synaesthetic colours. The stimuli used to test controls (i.e., the two graphemes and the original RGB triplets for rivalrous colours, prior to individual eye-dominance test) were matched to their own respective paired synaesthetes. All other experimental parameters remained identical to Experiment 1.

### 4.2 Results

**Fig. 2:**
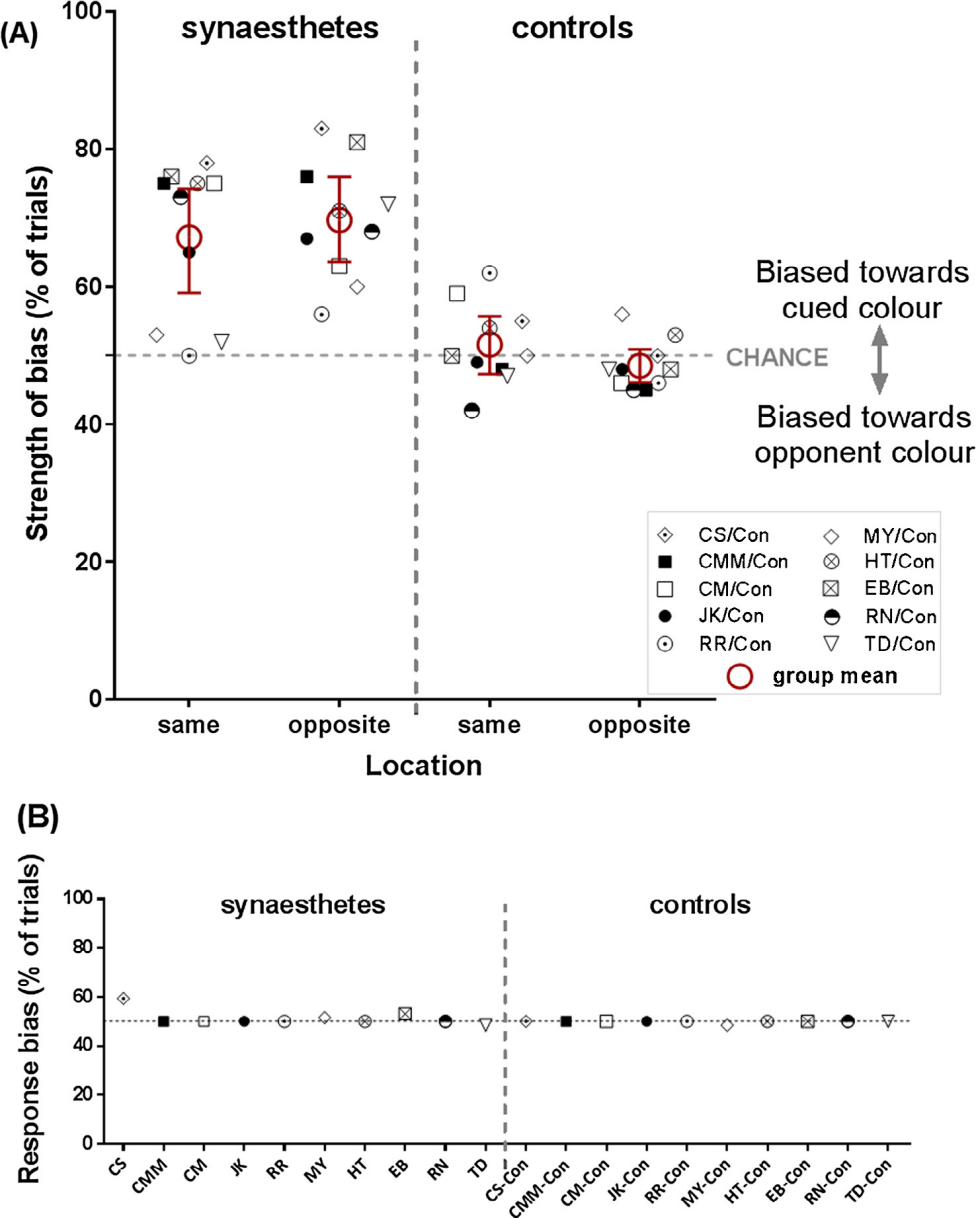
*Experiment 2: the effect of synaesthesia on binocular rivalry*. **(A)** Individual data and group averages for 10 synaesthetes (left of graph) and age-, sex- and language-matched non-synaesthetic controls (right of graph) showing the strength of perceptual bias in each condition, separated by cue-target location mappings. Values above/below chance indicate priming/suppression respectively. Synaesthetes again show priming from synaesthetic colour in both the same and opposite locations. There are no such effects in the controls. Error bars represent 95% confidence intervals around the mean. **(B)** Each data point represents the non-perceptual decisional/response bias for each participant in the mock-rivalry catch trials, plotted separately for the synaesthetes (left) and controls (right). Note that synaesthetes CM, CMM, JK, & MY previously participated in Experiment 1; CS, EB, HT, RN, RR & TD were new to Experiment 2. We use consistent symbols for each individual throughout the paper. (For interpretation of the references to colour in this figure legend, the reader is referred to the web version of this article.)

Data from the synaesthetes with the 0.25-s cue replicated the priming effects from Experiment 1, and this effect was specific to synaesthetes; there is no evidence of systematic priming or suppression in the controls (Fig. 2A). We again start by checking the extent to which the grey letter cues (which induce a colour experience for synaesthetes) influences subsequent binocular rivalry. Compared to chance, synaesthetes were again biased to see the cued colour for both locations (same location: 67.20%, *t*_(9)_ = 4.83, *p* = .001, *M_diff_* = 17.20, CI [9.14, 25.26], *d_unbiased_* = 1.40; opposite location: 69.70%, *t*_(9)_ = 7.13, *p* < .001, *M_diff_* = 19.70, CI [13.45, 25.95], *d_unbiased_* = 2.06).^1^ The control data, in contrast, showed no evidence of priming or habituation in either location (same location: 51.60%, *t*_(9)_ <1, *M_diff_* = 1.60, CI [−2.65, 5.85], *d_unbiased_* = 0.25; opposite location: 48.50%, *t*_(9)_ = −1.32, *p =* .22, *M_diff_* = −1.50, CI [−4.07, 1.07], *d_unbiased_* = 0.38).

A mixed-factor ANOVA with the factors of group (synaesthetes, controls) and location (same, opposite) confirmed robust priming in synaesthetes with no evidence for priming in controls (Fig. 2A; main effect of group: *F*_(1,18)_ = 35.27, *p* < .0001, *MoE_diff_* [synaesthetes-controls] = 6.46, *s_p_* = 6.88, CI [11.98, 24.90], *d_unbiased_* = 2.57); no main effect of location (*F*_(1,18)_ < 1, n.s., *M_diff_* [opposite-same] = 5.07, *s_p_* = 5.39, CI [−5.24, 4.90]); nor group × location interaction *F*_(1,18)_ = 2.62, *p* = .12, *MoE_diff_* [synaesthetes_(opposite-same)_ – controls_(opposite-same)_] = 7.27, *s_p_* = 7.74, CI [−1.61, 12.93]). The individual data (shown in Fig. 2A) show most of the synaesthetes follow the group pattern with data falling on the priming side above chance in both the same and opposite location conditions, whereas the controls cluster around chance. Fig. 2B shows the decision bias based on catch trials for each participant; only CS shows evidence of potential bias (approaching our criterion for exclusion: 59.38%).^2^

Experiment 2 replicated our initial priming effect from synaesthetic colour and demonstrated that this priming was specific to synaesthetes. We again see significant priming at both locations, suggesting the correspondence of cue-target locations is not important for synaesthetic colour to drive facilitatory bias. By using a shorter cue duration (0.25 s), we replicated this synaesthetic effect under conditions known to be insufficient for the slow and effortful voluntary mental imagery to trigger priming (Pearson et al., 2008).

## 5 Experiment 3

**Fig. 3:**
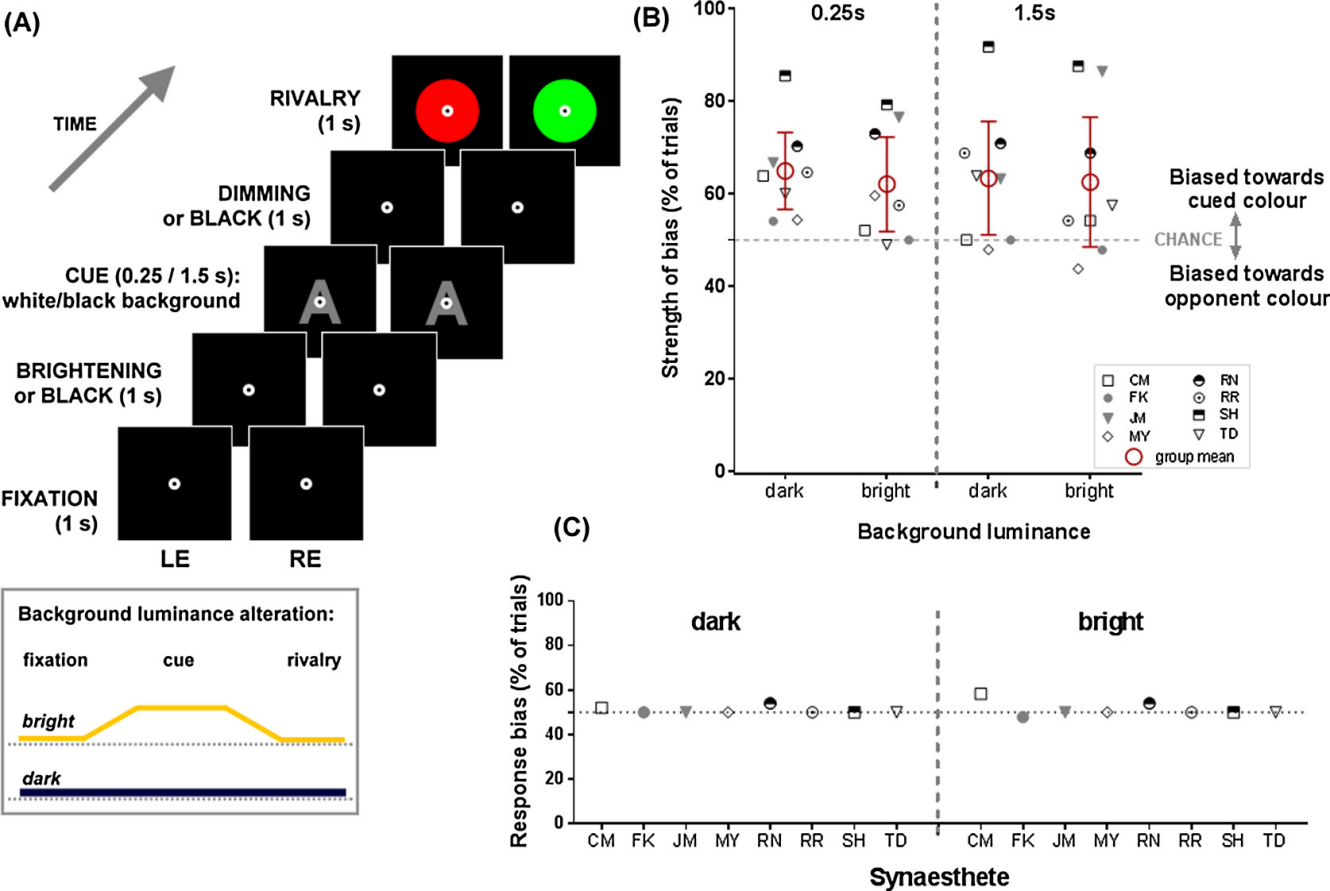
*Experiment 3: the effect of background luminance on the synaesthetic priming effect.***(A)** time-line of trial events of the bright and dark conditions (not to scale; see text for details). The alteration of background luminance over trial timeframe is illustrated in the lower box. **(B)** Data from 8 synaesthetes showing the strength of perceptual bias of each condition, grouped by different durations of the cue and background luminance (dark, bright). Error bars represent 95% confidence intervals. **(C)** Each data point represents response bias for each individual in the mock-rivalry catch trials, plotted separately for the dark (left) and bright (right) backgrounds.

In Experiment 3, we further investigated the nature of synaesthetic colour relative to voluntary imagery by manipulating concurrent uniform sensory stimulation. Previous research has shown that increasing background luminance disrupts the priming of mental imagery on subsequent binocular rivalry, which has been inferred to result from a disruption to the build-up of the mental image (Chang et al., 2013, Keogh and Pearson, 2011, Keogh and Pearson, 2014, Pearson et al., 2008, Sherwood and Pearson, 2010). If synaesthesia mechanistically resembles voluntary colour imagery, luminance should analogously interfere with synaesthetic priming. Here we manipulated the background luminance behind a centrally presented synaesthetic cue (Fig. 3A) to examine whether a luminance change during the cue would be as similarly disruptive to synaesthetic colour as to imagined colour. The cue duration was manipulated to see whether longer exposure to elevated luminance signals produced differential disruption to the synaesthetic effect.

### 5.1 Design

The procedure and design were identical to the previous experiments, with modifications specified below. We tested 8 synaesthetes (4 of whom participated in either Experiment 1 or 2; 2 of whom participated in both; see Table S1). They completed 3 blocks of 80 trials. In each block, we independently manipulated two experimental factors: duration of grapheme cue (0.25 s vs. 1.5 s) and background luminance (bright vs. dark). As illustrated in Fig. 3A, each trial began with a fixation display (1 s). In the bright condition, the luminance level of the background ramped up over one second to full brightness (61.74 cd/m^2^); a grapheme cue was then presented centrally on the full luminance background for either 0.25 s or 1.5 s, followed by 1 s in which the background luminance ramped down from full brightness to black (0.01 cd/m^2^). This was followed by the rivalry target, presented on a black background (1 s). As in the previous experiments, participants reported their percept during the binocular rivalry presentation. The dark condition had identical timeframe and visual parameters to the bright condition except that the background remained black throughout the entire trial. Each block of 80 trials consisted of 64 rivalry trials (80%) and 16 catch trials (20%). Summed across the three blocks, there were 192 true rivalry trials (hence, 48 trials per condition in this 2 × 2 factorial design) and 48 catch trials.

### 5.2 Results

The data (Fig. 3B) suggest that neither the presence of background luminance nor the different cue durations strongly influenced the synaesthetic colour priming effect, although there seems greater uncertainty in the long duration conditions (larger confidence intervals). Checking each condition relative to chance showed significant priming when the cue was shown for 0.25 s on either a dark background (*t*_(7)_ = 4.22, *p* = .004, *M_diff_* = 14.88, CI [6.53, 23.22], *d_unbiased_* = 1.33) or bright background (*t*_(7)_ = 2.77, *p* = .03, *M_diff_* = 12.00, CI [1.77, 22.23], *d_unbiased_* = 0.87). There was also significant priming when the cue was presented for 1.5 s on a dark background (*t*_(7)_ = 2.58, *p* = .04, *M_diff_* = 13.38, CI [1.12, 25.63], *d_unbiased_* = 0.81), and no clear statistical outcome when the cue was shown for 1.5 s on a bright background, despite a trend of priming (*t*_(7)_ = 2.11, *p* = .07, *M_diff_* = 12.50, CI [−1.53, 26.53], *d_unbiased_* = 0.66). The confidence intervals illustrate greater uncertainty in the long duration conditions, but the short duration conditions clearly replicate Experiments 1 and 2, regardless of background luminance. Overall, synaesthetic inducers again drove a facilitatory bias towards seeing cued colour in binocular rivalry, replicating our previous experiments. We see little evidence for priming or suppression in the non-rivalry catch trials (Fig. 3C), consistent with little, if any, influence of response bias. In the bright condition, CM showed some minor bias, approaching our criterion (58.3%).^3^ The minimal effect of luminance is distinct from voluntary imagery, which is sensitive to perturbation from background luminance (Chang et al., 2013, Pearson et al., 2008, Sherwood and Pearson, 2010).

The data from the first three experiments suggest that the priming effect of synaesthetic colour on conscious vision is relatively invariant to changes in low-level visual properties such as retinotopic location and concurrent luminance stimulation/alternation, both largely coded in the early visual cortices. This argues for a relatively late representational locus in the cascade of visual processing for synaesthetic colour, compared to actual colour and imagined colour, which involve early visual mechanisms from retina to primary visual regions (Bergmann et al., 2015, Chang et al., 2013, Naselaris et al., 2015, Pearson et al., 2008; for a review of relevant literature, see Pearson, Naselaris, Holmes, & Kosslyn, 2015).

## 6 Experiment 4A & 4B

There is good evidence that attention to a synaesthesia-inducing alphanumeric stimulus is a critical factor for eliciting synaesthetic colour. Under conditions where attention is diverted from an inducer, the impact of synaesthetic colour diminishes or disappears (e.g., Mattingley et al., 2006, Rich and Mattingley, 2010). In Experiment 4, we manipulated the amount of attention available for processing the inducing grapheme by introducing a demanding concurrent task designed to divert attention from either the colour-inducing grapheme (Experiment 4A: colour formation) or from the maintenance of the synaesthetic colour (Experiment 4B: colour storage).

### 6.1 Design

**Fig. 4:**
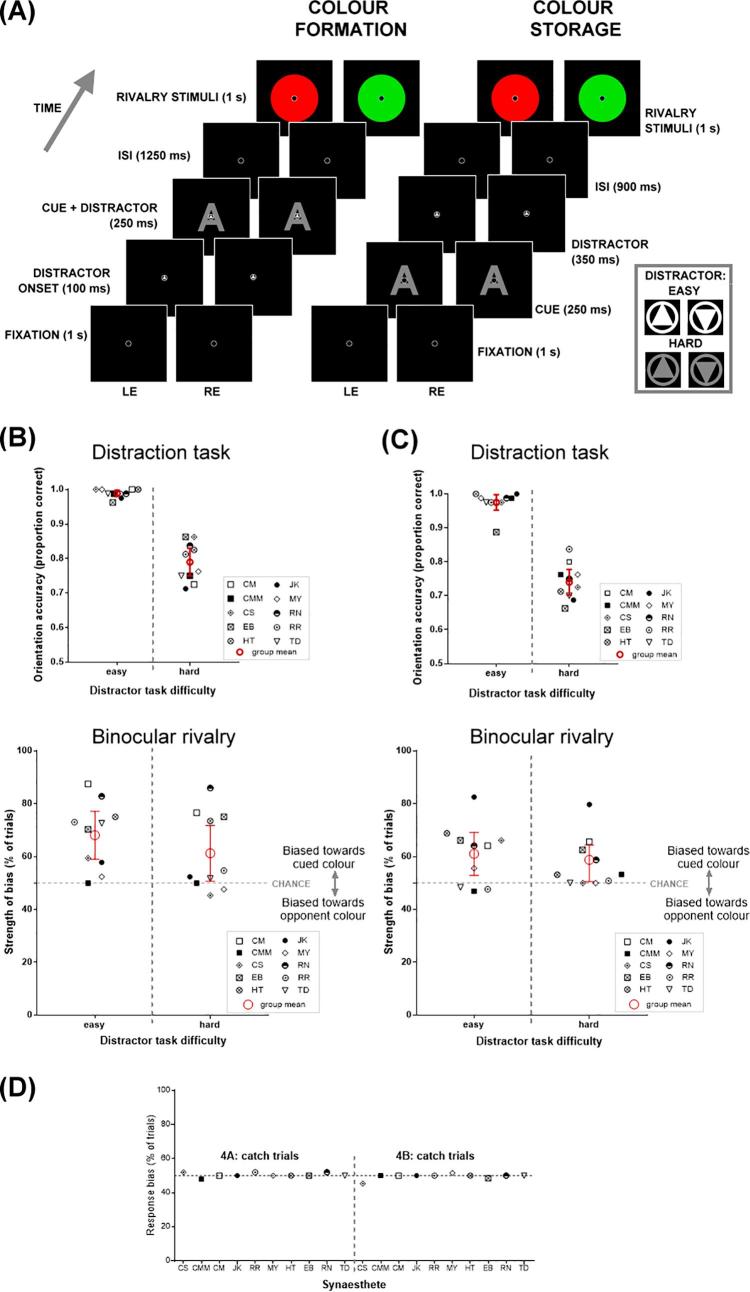
*Experiment 4A and 4B: The effect of attention on the synaesthetic binocular rivalry effect.***(A)** Timeline of trial events of the synaesthetic colour formation (left) and synaesthetic colour storage (right) experiments. Example stimuli of the distraction task are illustrated in the box (far right), separated by orientation and difficulty (note that in the hard condition the contrast of the triangle varied with performance). **(B)** Experiment 4A Colour Formation data. *Upper*: Mean accuracy of distraction task performance, plotted by difficulty level. *Lower*: The strength of perceptual bias during binocular rivalry, separated by difficulty levels in the distraction task. Error bars show 95% confidence intervals. **(C)** Experiment 4B Colour Storage data*. Upper*: Mean accuracy of distraction task performance, plotted by difficulty level. *Lower*: The strength of perceptual bias during binocular rivalry, separated by difficulty levels in the distraction task. Error bars show 95% confidence intervals. **(D)** Response bias calculated from catch trials in the binocular rivalry task. Left: Experiment 4A; Right: Experiment 4B. (For interpretation of the references to colour in this figure legend, the reader is referred to the web version of this article.)

The main testing procedure and design were identical to the previous experiments, with modifications specified below. We tested 10 synaesthetes (all of whom had participated in at least one other experiment, see Table S1), and within a single session they first completed Experiment 4A and then Experiment 4B. In Experiment 4A (distraction during synaesthetic colour formation), the participants completed two blocks of 80 trials. Each trial began with a fixation display (1 s, see Fig. 4A left), followed by a distractor stimulus (a triangle pointing upwards or downwards) appearing inside the fixation circle (0.1 s). This was succeeded by a display simultaneously presenting a graphemic cue with the triangular distractor persisting (0.25 s; the distractor was hence shown for 0.35 s in total), followed by an ISI of 1.25 s, and finally the rivalry stimulus (1 s). We presented the triangle first to engage attention to the triangle task prior to the presentation of the inducer. Participants first reported the dominant colour they perceived during the rivalry presentation using their right hand to press the designated key and then pressed another designated key using their left hand to report the orientation of the distractor. Each block of 80 trials was composed of 64 rivalry trials (80%) and 16 catch trials (20%). Summed across the two blocks, there were 128 true rivalry trials in total and 32 catch trials.

Experiment 4B (distraction during storage, see Fig. 4A right) had a similar design and the same task requirement as Experiment 4A, except that the graphemic cue (0.25 s, without any accompanying distractor) was presented immediately after the initial fixation and was then followed by the distractor (a triangle embedded within the fixation circle, without a simultaneous grapheme) for 0.35 s. The proportion of catch and rivalry trials was identical to Experiment 4A.

In both Experiment 4A and 4B, there were two levels of difficulty randomly interleaved within each block of trials. In the easy condition, the distractor was always a white triangle (61.74 cd/m^2^) on a black background (0.01 cd/m^2^); the high contrast made the stimulus highly visible. In the hard condition, the distractor was a grey triangle on a black background; the lower contrast made the stimulus less visible. Further, the triangle’s brightness in the hard condition was varied on a trial-by-trial basis using a staircase procedure that dynamically altered the contrast of the triangles. The initial brightness of the distractor was set at 0.85 cd/m^2^; if the participant had two consecutive correct responses on the distraction task (i.e., two correctly reported orientations), the stimulus luminance, and therefore its contrast with regard to the black background, was reduced; if the participant made an erroneous response, the stimulus was made brighter. With this interactive procedure, we ensured that in the hard trials, the distraction task was kept challenging and demanding enough to divert attention away from the cue. Note that while the concurrent distraction task was designed to decrease attention available to process the inducing grapheme, we have no measure of the remaining attentional resources. While unlikely to completely prevent the processing of the inducer, the ‘hard’ condition should result in less attention to the grapheme relative to the ‘easy’ distractor task, which means we can examine the impact of this on the synaesthetic priming effect. In each block, half of the trials were easy trials, while the remaining half were hard trials, randomly intermingled. As this dual-task procedure could be difficult initially, our participants were given 32 practice trials before the main experiments.

### 6.2 Results

In Experiment 4A, performance on the attentional distraction task was poorer in the hard (79.00%) than in the easy condition (98.88%, *t*_(9)_ = 10.64, *p* < 0.00001, *M_diff_* = 19.88, *s_diff_* = 5.91, CI [15.65, 24.10], *d_unbiased_* = 4.44; see Fig. 4B upper graph). Note that even in the hard condition, performance was still almost 80% correct, suggesting that although we have increased the difficulty of the task, it probably caused only a relatively small reduction in the available attentional resources to process the cue. Despite this, we do see modulation on the magnitude of rivalry priming (albeit a small one). There was less priming in the hard (61.26%) compared to the easy condition (68.08%; *t*_(9)_ = 2.42, *p* = .04, *M_diff_* = 6.82, *s_diff_* = 8.90, CI [0.46, 13.18], *d_unbiased_* = 0.45; see Fig. 4B lower). In both conditions, we replicate our synaesthetic binocular rivalry priming effect (comparison to chance: easy: *t*_(9)_ = 4.45, *p* = .002, *M_diff_* = 18.10, CI [8.90, 27.30], *d_unbiased_* = 1.30; hard: *t*_(9)_ = 2.42, *p* = .04, *M_diff_* = 11.30, CI [0.73, 21.87], *d_unbiased_* = 0.70). The confidence intervals show greater uncertainty of synaesthetic priming on rivalry in the hard condition. Fig. 4D (left) presents the response bias for the catch trials for each individual, showing no evidence of systematic bias.

In Experiment 4B, for the attentional distraction task, performance was again poorer in the hard (74.00%) than the easy (97.50%) condition (*t*_(9)_ = 14.39, *p* < .000001, *M_diff_* = 23.50, *s_diff_* = 5.16, CI [19.81, 27.19], *d_unbiased_* = 4.86; see Fig. 4C upper). Unlike Experiment 4A, this small manipulation of attention during the delay between inducer and rivalry stimuli did not have a clear effect on binocular rivalry. Binocular rivalry bias was 61.01% for the easy condition and 57.37% for the hard condition, which did not differ statistically (*t*_(9)_ = 1.53, *p* = .16, *M_diff_* = 3.64, *s_diff_* = 7.51, CI [-1.74, 9.01], *d_unbiased_* = 0.32; see Fig. 4C lower). We cannot determine whether this reflects that attentional manipulation during storage has no effect, whether we needed a stronger attentional manipulation, or whether there is differential priming that we lack the power to detect. In both the easy and hard conditions though, we again replicated the basic effect of synaesthetic priming on binocular rivalry, relative to chance (easy: *t*_(9)_ = 3.07, *p* = .01, *M_diff_* = 11.10, CI [2.93, 19.27], *d_unbiased_* = 0.89; hard: *t*_(9)_ = 2.42, *p* = .038, *M_diff_* = 7.50, CI [0.50, 14.50], *d_unbiased_* = 0.70). Fig. 4D (right) presents the response bias for catch trials, again showing no systematic bias that could drive the results.

These data show that attention influences the synaesthetic binocular rivalry effect during the generation of the anomalous colour percept. Participants were still able to perform well in the ‘hard’ condition of our attention task, but even this small reduction in available attentional resources attenuated the effect of synaesthesia on binocular rivalry. Interestingly, manipulating attention seems to have opposite effects on normal and synaesthetic colour. Specifically, Suzuki and Grabowecky (2003) measured afterimage durations and their time of onset/offset while participants were asked to either focus attention to a colour patch that served to habituate vision and induce an afterimage or turn attention away from the colour patch. When attention was turned away from the ‘inducer’, the afterimage appeared earlier and disappeared later (longer-lasting afterimage, suggesting a stronger habituation effect), compared to when attention was focused on the inducer. This pattern is the opposite of what we see here with synaesthetic colour – when attention is turned away from a synaesthetic inducer, the synaesthetic effect is weaker than when attention is focused on the inducer.

## 7 Experiment 5

In the final experiment, we examined the effect of synaesthetes’ voluntary colour imagery on binocular rivalry. We looked specifically at two aspects. First, whether synaesthetes show different imagery strength relative to controls, using the rivalry paradigm as our measure; second, whether imagery priming in synaesthetes is location-specific; that is, whether it occurs only when imagined and perceived colour overlap retinotopically as it does with non-synaesthetes (Bergmann et al., 2015, Chang et al., 2013, Pearson et al., 2008). The results of Experiments 1 and 2 suggest the synaesthetic priming of binocular rivalry is not limited to the same retinotopic location. This could be because the synaesthetic colour induced is more diffuse (or just large enough to cover both rivalry stimulus locations), or because endogenous, higher-level visual priming (be it synaesthetic or imagined) for synaesthetes is generally less retinotopically-constrained compared to non-synaesthetes. Note that Experiment 1 showed that synaesthetes have the typical patterns of retinotopically-constrained habituation from ‘normal’ colours, replicating the local adaptation seen in non-synaesthetes.

### 7.1 Design

We tested 6 synaesthetes and compared them to two separate sets of non-synaesthetic controls. To assess whether synaesthetes and controls differ in the strength of voluntary imagery priming, we tested a Demographic-match group: 12 controls (2 for each synaesthete) matched based on sex, age, and language. Two controls had to be excluded from the analysis of binocular rivalry results – one failed to understand the instructions, and the other reported > 40% of ‘mixture’ in true rivalry trials, giving too few dominant percepts for group analysis, leaving datasets of 10 controls. To examine whether voluntary imagery effects in synaesthetes are retinotopically constrained as in non-synaesthetes it is important to match their baseline imagery result (i.e., priming at the same-location condition, in which the locus of imagined colour and rivalry target was identical), hence we also tested an Imagery-match control group. We tested 30 new non-synaesthetic controls of whom 12 were selected whose strength of imagery priming in the same-location condition was within ±5% from that of a given paired synaesthete (except Control F, whose priming was +10% from the paired synaesthete), with an additional criterion of not being lower than chance level. Participants in this Imagery-matched group were not matched to synaesthetes on demographic traits. Note that the criterion of identifying participants (i.e., ± 5%, priming ≥ 50% in the same condition) was independent of the effect of interest (whether synaesthetes and controls differed at the opposite location).

**Fig. 5:**
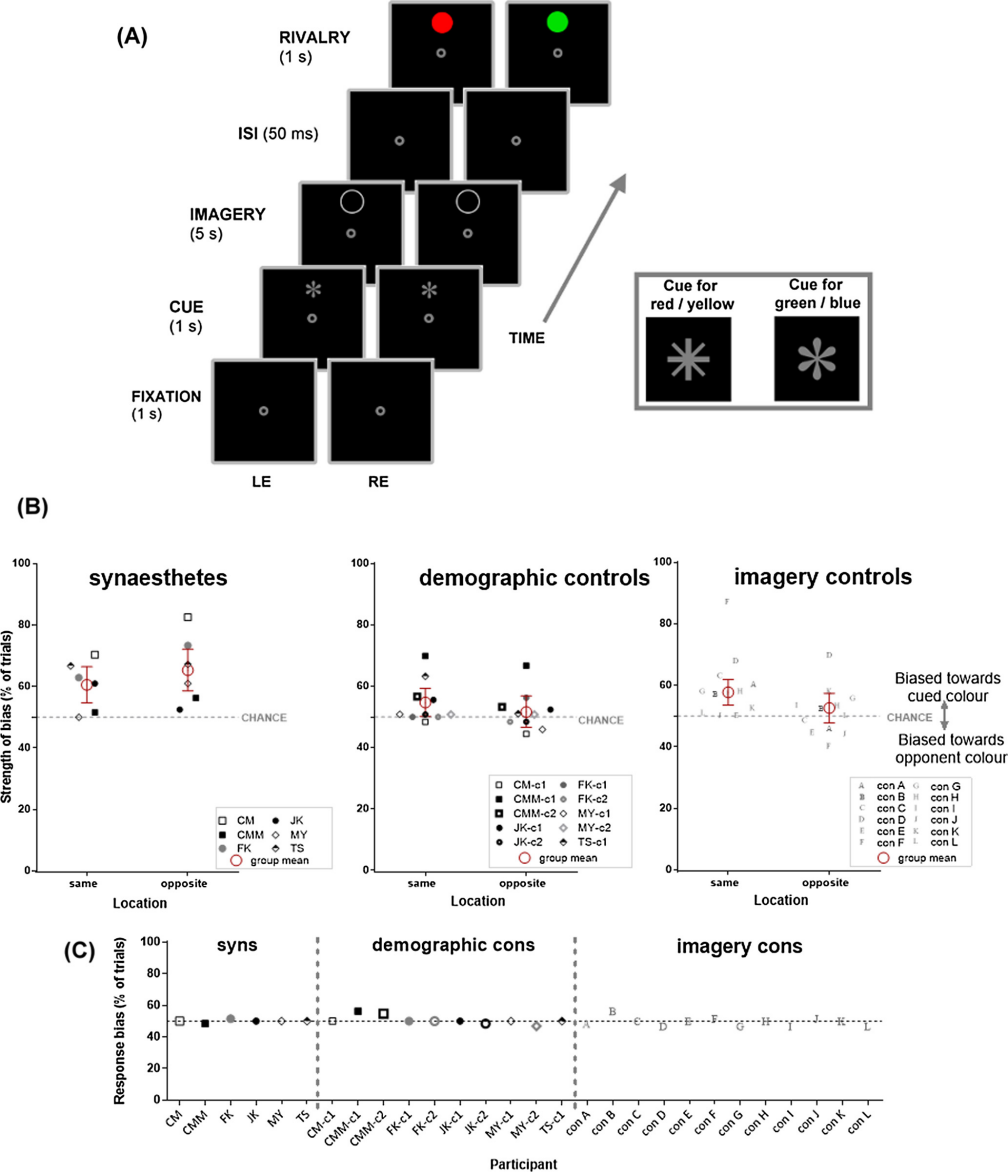
Experiment 5: Voluntary imagery effects on binocular rivalry. **(A)** Time-line of trial events. Inset box (right) shows the geometric symbols (cues) prompting imagery of different colours. Note that cues and rivalry targets could appear in the same or opposite locations (same location shown here). **(B)** Data from 6 synaesthetes (left), 10 controls matched based on demographics (demographic controls; middle), and 12 controls matched based on priming magnitude of the same condition (imagery controls; right). The strength of perceptual bias of each condition is plotted as a function of the different groups of participants and location. Error bars are 95% confidence intervals. **(C)** Response bias calculated from catch trials in the binocular rivalry task for each of the groups.

Participants completed 2 blocks of 80 trials, including catch trials, using proportions identical to Experiment 1, where we also manipulated spatial compatibility (same vs. opposite). We modified the timeframe of trial events and used two different geometric symbols as the cues that instructed participants which colour to imagine, instead of graphemic or normal colour cues. This was to avoid directly inducing synaesthetic colour by presenting letter or word cues. As Fig. 5A shows, each trial began with a fixation point (1 s), followed by a geometric symbol cue (1 s, see the inset box for illustration). Following the cue was a 5 s interval during which a grey circle (diameter: ∼2.7°; the interior being black) was presented either 2.6° above or below the centre of the fixation point. We asked participants to engage in active visual imagery, filling the circle with the prompted colour during the 5 s period. This was followed by a 50-ms ISI and 1-s presentation of binocular rivalry. Participants were given 5 s to respond. After an ITI of 1.5 s, the next trial began. Prior to the experiments, they had 1 practice block of 15 trials. All of the participants filled in the Vividness of Visual Imagery Questionnaire (VVIQ) after they had completed the binocular rivalry experiment.

### 7.2 Results

We first compared the subjective ratings of imagery vividness for the synaesthetes with the Demographic-match control group. Note, for this comparison the data of all 12 demographic-match controls were included. Synaesthetes (mean = 64.00, *SD* = 8.81) scored higher than demographic-match controls (mean = 54.00, *SD* = 8.34; *t*_(14)_ = 2.36, *p* = .03, *MoE_diff_*[synaesthetes-controls] = 9.00, *s_p_* = 8.49, CI [1.00, 19.00], *d_unbiased_* = 1.12). Due to the large confidence intervals, we do not wish to place too much emphasis on this difference, but it is consistent with other synaesthesia literature that reports more vivid imagery in synaesthetes using the VVIQ (Barnett & Newell, 2008). The imagery-matched control group, selected on the basis of rivalry bias data for the same-location condition also completed the VVIQ. The VVIQ mean of this group was 56.25 (SD = 10.87).

Next, we looked at the priming data of the binocular rivalry experiment (Fig. 5B). As we selected the Imagery-matched group of controls to approximately match synaesthetes in the magnitude of priming in the ‘same’ condition, we could not analyse all three groups together. Instead, we first compare synaesthetes and the demographically-matched controls to test for overall effects of voluntary imagery on binocular rivalry, by location. We then compare the synaesthetes with the imagery-matched controls in only the opposite location to specifically test whether voluntary colour imagery is unusually unconstrained by retinotopic location in our synaesthetes.

A mixed-designed ANOVA with the factors of group (synaesthetes, demographic-match controls) and location (same, opposite) on rivalry biases revealed a significant interaction between group and location (*F*_(1,14)_ = 6.29, *p* = .03, *MoE_diff_* [synaesthetes_(opposite-same)_ - demographic-match controls_(opposite-same)_] = 6.65%, *s_p_* = 6.01, CI [1.26, 14.56], *d_unbiased_* = 1.25). Breaking the interaction down by location showed that, in the *same* location, we have no evidence that the two groups differ in their strength of priming (*MoE_diff_* [synaesthetes- demographic-match controls] = 8.20, *s_p_ =* 7.40, CI [−2.42, 13.98], *p* = .15). In the *opposite* location, synaesthetes show greater imagery priming than the controls (*MoE_diff_* [synaesthetes- demographic-match controls] = 9.30, *s_p_* = 8.40, CI [4.39, 22.99], *p* = .007, *d_unbiased_* = 1.54). However, interpreting these results is complicated by the fact that the demographically-matched control group failed to show strong imagery priming even in the *same* location (relative to chance: *t*_(9)_ = 2.13, *p* = .06, *M_diff_* = 4.70, CI [-0.30, 9.70], *d_unbiased_* = 0.61). By contrast, synaesthetes showed priming greater than chance at both locations (*same*: *t*_(5)_ = 3.21, *p* = .02, *M_diff_* = 10.50, CI [2.08, 18.92], *d_unbiased_* = 1.10; *opposite: t*_(5)_ = 3.28, *p* = .02, *M_diff_* = 15.33, CI [3.30, 27.37], *d_unbiased_* = 1.13). Thus, synaesthetes show priming at both locations, while this group of controls showed little priming.

Our test with the imagery-matched controls is a stronger test of whether synaesthetes have unusual effects of imagery in the *opposite* location, given that the two groups were selected to have comparable degree of priming in the *same* condition. Synaesthetes showed greater priming than the imagery-matched controls at the *opposite* location (independent-measures *t*-test: *t*_(16)_ = 3.15, *p* = .006, *MoE_diff_* [synaesthetes- imagery-match controls]= 9.57, *s_p_* = 9.03, CI [4.66, 23.80], *d_unbiased_* = 1.50). As the imagery-matched controls were selected to have some indication of priming in the *same* location condition, it would be circular to analyse this, we can only document that they failed to show evidence of priming in the *opposite* location (*t*_(11)_ = 0.51, *p* = .62, *M_diff_* = 1.17, CI [−3.89, 6.23], *d_unbiased_* = 0.14).

Together, the results of Experiment 5 suggest that imagery in synaesthetes may be stronger, more vivid and global as compared to non-synaesthetes. Synaesthetes’ voluntary imagery effect on binocular rivalry seems to differ from controls in the degree of constraint by retinotopic locations, even when we controlled for imagery strength. The results from non-synaesthetic controls are weaker than previously published work on the effect of pure colour imagery on binocular rivalry (Chang et al., 2013), which may reflect the documented individual differences in location and spatial orientation tuning that is associated with the size of primary visual cortex (Bergmann et al., 2015). However, synaesthetes showed priming regardless of same or opposite location. One possible explanation for this ‘global’ effect is that, despite using non-inducing cues, synaesthetes might subvocally name the colour to be imagined, which could then evoke some synaesthesia. Thus, synaesthetes could be showing a combination of a non-constrained synaesthetic effect (as in Experiments 1 and 2) with a retinotopic-specific voluntary imagery effect. Alternatively, synaesthetes may have unusual voluntary colour imagery due to possible differences at the neurophysiological level. We expound on this in the Discussion.

## 8 Discussion

Here we devised a novel approach to study synaesthetic colour, using binocular rivalry to probe its functional impact on subsequent conscious vision and compare it to other types of colour experiences. We find that normal colour and synaesthetic colour have opposite effects on subsequent conscious vision, despite apparent phenomenological equivalence. Exposure to normal colour causes chromatic adaptation, a decrease in neural sensitivity of colour-sensitive neurons to the adapted colour. This results in a suppressive after-effect such that colour vision is more responsive to the opponent colour in subsequent rivalry displays (McCollough, 1965, Webster, 2011). Synaesthetes exhibited this typical effect (Experiment 1). By contrast, inducing synaesthetic colour resulted in facilitatory priming, similar to the effects of imagined colour (Chang et al., 2013, Pearson et al., 2008), with a crucial difference. In control participants, priming from imagery is constrained by retinotopic visual space (Chang et al., 2013, Pearson et al., 2008). Priming from synaesthesia, however, occurred both when the cue and rivalry stimulus appeared at the same and different locations. This location-independent priming might be due to synaesthetic colours being diffuse across the visual field, rather than limited to the inducer’s location. Another possibility is that synaesthetic colours appear in a separate reference frame from the physical visual field (e.g., in the mind’s eye) and exert influences that are not bound to physical location. Finally, it is possible that although synaesthetic colours appear locally at the inducing grapheme’s location, their effects can spread outside to other locations. Future research is necessary to distinguish these potential mechanisms. Regardless, our results clearly indicate that synaesthesia reflects a unique psychophysical profile – similar to high-level imagined colour in the direction of functional impact (facilitatory bias/priming), but unlike its location-specificity with regard to the inducing stimulus, and distinct from low-level ‘normal’ colour in both direction (priming vs. habituation) and location-specificity (global vs. local).

Synaesthetic priming was replicated across four experiments, involving 14 different synaesthetes, and different durations (0.25 s & 5 s).^4^ It is specific to synaesthetes – the same paradigm showed no perceptual bias in non-synaesthetic controls – and is relatively unaffected by low-level sensory manipulations, such as background luminance and retinotopic location, which typically disrupt visual priming from voluntary colour imagery (Keogh and Pearson, 2017, Pearson et al., 2008, Pearson et al., 2015). Further, we see little evidence of response bias, as measured by our catch trials. Although response bias is always a tricky issue in binocular rivalry studies, the reliable results across experiments, and the addition of catch trials, reduces the likelihood that our results are driven by response bias. Consistent with other synaesthesia literature, reducing attention to the inducing cue through a difficult concurrent task attenuated the synaesthetic priming effect. As with other studies manipulating attention away from the inducing stimulus (e.g., Mattingley et al., 2006, Sagiv et al., 2006), our attentional manipulation reduced, but did not eliminate synaesthetic effects, perhaps because even in our ‘hard’ condition participants were still able to perform the attention task at a relatively high accuracy (>74%).

The unique psychophysical profile of synaesthetic priming gives clues as to where such unusual colour experiences might arise in the brain. Significant synaesthetic priming found at both same and opposite locations suggests that the physical location of an inducing grapheme has minimal influence on subsequent priming, a striking difference from normal and imagined colour. As discussed earlier, this global priming might result from either synaesthetic colour spreading across multiple parts of visual field (i.e., still appearing in the same reference frame as binocular rivalry stimuli but having broader spatial coverage) or synaesthetic colour existing in a separate reference frame and affecting binocular perception in a location-independent fashion (e.g., appearing in the mind’s eye as an abstract entity of ‘redness’, without exact locus and contour). Exploring whether these possibilities have any bearing on the priming effect would be an interesting future direction. Importantly, our data clearly show that the synaesthetic priming effect is not constrained by the inducer location in the receptive field (i.e., upper vs. lower location), whereas normal colour is strongly confined by the location of the colour patch in the receptive field (also see evidence of imagery being similarly constrained by retinotopic correspondence, see Chang et al., 2013, Pearson et al., 2008, Slotnick et al., 2005). Thus, our results, together with prior literature of normal and imagined colour, are consistent with the idea that different colour experiences are constrained by different forms of spatial reference frames.

There is good evidence that higher-level visual areas, such as those specialised for perceiving faces and scenes, show invariance to transformation of lower-level visual properties such as visual location (Schwarzlose, Swisher, Dang, & Kanwisher, 2008), size (Eger, Schyns, & Kleinschmidt, 2004), and spatial orientation (Axelrod & Yovel, 2012). In particular, the invariance to visual location is associated with coding of complex stimuli via the amalgamation of neural activity at multiple lower-level regions, culminating in a representation beyond the scope of retinotopically-based regions (Grill-Spector & Weiner, 2014). Akin to the global priming that we observe with synaesthetic colour, high-level visual areas respond to their preferred stimuli regardless of location in the visual field; they are sensitive to category/identity, but indifferent to location. This resemblance suggests that either synaesthetic colour is represented in retinotopic visual cortices but additionally involves cortical sections outside the inducer’s corresponding retinotopic area or, perhaps more plausibly, that the core neural substrates of synaesthetic colour are beyond the realm of retinotopic cortex, perhaps in high-level regions for semantic knowledge about object colour (e.g., the anterior temporal lobe, see Chiou and Lambon Ralph, 2016, Chiou and Rich, 2014, Chiou et al., 2014).

While there are multiple cortical subregions of the ventral occipitotemporal cortex that are colour-sensitive, evidence from both fMRI and single-unit recording has shown that area V4 is particularly crucial for maintaining colour constancy across various illuminant conditions, making colour perception immune to changes of local spectral composition (Bannert and Bartels, 2017, Foster, 2011). Its pivotal role in normal colour perception has led synaesthesia researchers to examine if it similarly underlies synaesthetic colour. Although some studies claim V4 activation is driven by synaesthesia (e.g., Hubbard et al., 2005), these findings have been challenged on methodological grounds (Chiou and Rich, 2014, Hupé et al., 2012). It is well-established that V4 is retinotopic, with colour presented at an upper/lower retinal location being mapped to the lateral/medial side of V4 (e.g., McKeefry and Zeki, 1997, Wade et al., 2002). Our priming at a different location from the cue might therefore be inconsistent with the strong claim that V4 is involved in representing synaesthetic colour, at least if one assumes our global effect reflects a lack of constraint to retinotopically-mapped locations. It is possible that synaesthetic colour exists in the same retinotopically-based reference frame as that of the physical visual world (but appears more diffuse). Given the descriptions of most synaesthetes, however, it seems more plausible that its reference frame is completely separable from the visual world and does not obey the retinotopic laws. This raises the possibility of separate neural origins for different types of colour experiences – normal (perceptual) and imagined colour are represented in lower stages of processing, involved in *perceiving* colour (Howard et al., 1998, Naselaris et al., 2015, Rich et al., 2006; for review about the role of early visual cortex in imagery, see Winlove et al., 2018), whereas synaesthetic colour could recruit higher regions involved in *knowing about* colour (for relevant discussion about synaesthesia resembling colour knowledge and 'ideathesia', see Chiou and Rich, 2014, Mroczko-Wąsowicz and Nikolić, 2014, Rich et al., 2006). We do not mean that regions ‘low’ in the processing hierarchy are unrelated to synaesthesia. They could still be affected by synaesthesia if it is primarily represented elsewhere. As we discuss later, this might provide some explanation for recent evidence of synaesthetes showing anomalies in sensory-level visual processing (e.g., Banissy et al., 2013, Terhune et al., 2011).

The robustness of synaesthetic priming to low-level sensory interference is consistent with observations that, for most synaesthetes, the appearance of inducers (e.g., font) has a negligible or no effect on synaesthesia (Chiou and Rich, 2014, Rich et al., 2005). By contrast, higher-level cognitive factors, such as grapheme recognition or subjective interpretation of an ambiguous symbol, strongly modulate synaesthetic colour (Bargary et al., 2009, Dixon et al., 2006, Mattingley et al., 2001). In light of multiple studies showing the potent influences of high-level factors, it is interesting to note that Nikolić, Lichti, and Singer (2007) reported evidence of colour opponency effects in synaesthesia: When a grapheme was shown in an opponent colour relative to the synaesthetic colour (e.g., ‘A’ induces red but is printed green) synaesthetes’ colour naming latency was further slowed compared to the ‘non-opponent’ incongruent condition (e.g., red-inducing ‘A’ printed in blue). It merits further investigation to know whether this opponency effect occurs at the stage of hue perception (e.g., red synaesthetic colour hinders the perception of green more than blue) or response selection (red synaesthetic colour interrupts the utterance of ‘green’ more than ‘blue’).

The reliance on higher level cognitive factors meshes with the effect of attentional diversion – for priming to occur, a synaesthete needs to attend to an inducer, recognise it, and access its lexical meaning and other associated attributes for the experience of synaesthetic colour to emerge. A distractor may reduce the amount of attention an inducer receives, which hinders all downstream cognitive and neural events that lead to synaesthetic colour. It is noteworthy that typical forms of semantic priming can be mediated by the conceptual association between linguistic entities, for example ‘nurse’ primes ‘doctor’. However, the synaesthetic priming here involves both semantic processing of the graphemic inducer and an additional, anomalous conscious experience of colour. Thus, by definition, it is not entirely equivalent to semantic priming.

It remains unknown what exactly induces synaesthetic priming and why voluntary imagery in synaesthetes is stronger, more vivid, but less retinotopically constrained. Although our data are unable to differentiate whether it is the graphemic cue eliciting priming directly or via the evoked colour, both alternatives are anomalous, and do not occur in control participants. The former explanation relies on the grapheme itself directly impacting rivalry, with synaesthetic colour being epiphenomenal. More plausibly, given that the only known phenomenon linking graphemic cues with colour rivalry (the colour) is the synaesthetic experience itself, we interpret these findings as evidence that the cue induces synaesthetic colour, and it is this colour that interacts with the sensory signals of colour rivalry that produces priming. Note that here both cues and rivalry targets were presented visually, it remains to be tested whether other cross-modal forms of synaesthesia (e.g., music induces colour, see Chiou, Stelter, & Rich, 2013) also affect binocular rivalry.

Regarding the unusual imagery characteristics in synaesthetes, there are (at least) two plausible explanations. First, it is possible that despite the geometric non-synaesthesia inducing cue symbols, the participants were actively rehearsing the relevant colour name and therefore evoking the related synaesthetic colour. Although we have no overt evidence of such rehearsal, it seems plausible. In this case, however, one might imagine that the effect in the same condition should be greater than in the opposite condition because of the summed effect of synaesthesia plus imagery, compared to synaesthesia alone (in fact, the pattern of data was, if anything, in the opposite direction, with greater priming in the *opposite* than the same location, see Fig. 5B). We hesitate to place much emphasis on this counter-argument, however, as our evidence of colour imagery priming on binocular rivalry in the same location was not strong in non-synaesthetic controls. Another possibility is that having synaesthesia may be related to a more excitable visual cortex, as Terhune et al. (2011) argue. Terhune et al. observed lower thresholds for TMS-induced phosphenes, which has been interpreted as evidence of hyper-excitability. In the present study, colour priming from both synaesthesia and imagery was not constrained to the retinotopic locations of the inducers, which could be seen as consistent with hyper-excitability. Interestingly, recent research has linked primary visual cortex excitability with imagery strength (Keogh, Bergmann, & Pearson, 2016), and other work has linked the extent to which imagery is local in visual space and its degree of rivalry priming with the size of primary visual cortex (Bergmann et al., 2015). It is worth emphasizing that the relationship between synaesthesia and cortical excitability remains speculative and further research is needed to clarify the relationship between visual cortex excitability and its size, and how they interact with mental imagery.

In sum, in this study we use a novel psychophysical technique to probe the nature of synaesthetic colour and explore its impact on conscious vision. With our approach, we identify the commonalities and distinctions between normal, imagined, and synaesthetic colour. The unique psychophysical profile of synaesthesia constrains theories about its neural basis and contributes to our general understanding of the different levels of representation in colour processing. Synaesthetic colour *subjectively* may resemble vivid normal colour (while not veraciously mimicking it), but *functionally* it behaves more like colour imagery that is not constrained to the inducer location, making it a unique experience.

## Author contributions

RC, JP, & ANR designed the study; RC & SR collected the data; RC, ANR, & SR analysed the data; RC, JP, ANR, & SR wrote the paper.

## Appendix A Supplementary material

Supplementary data 1 Supplemental Information includes supplemental analysis, example stimuli, and supplemental details about participation

Supplementary Data S2 This is open data under the CC BY license (http://creativecommons.org/licenses/by/4.0/).

## Footnotes

1 If we remove the 4 synaesthetes who also participated in Expt 1, the results still replicate (despite the small sample size of 6): Significant priming at the same location: 67.27%, *t*_(5)_ = 3.32, *p* = .02, CI: [56.98, 77.55], and in the opposite location: 71.97%, *t*_(5)_ = 5.49, *p* = .003, CI: [64.25, 79.68]; the same vs. opposite comparison is not significant: *t*_(5)_ = −1.22, *p* = .27, CI: [−2.48, 11.88].

2 Re-doing the analysis without this synaesthete does not alter the pattern of results: the main effect of group remains (*F*_(1,17)_ = 33.36, *p* < .0001).

3 Re-doing the analysis without this synaesthete for the bright condition does not alter the pattern of results: relative to chance there is a significant cueing effect when the cue was brief (*t*_(6)_ = 2.85, *p* = .03, *M_diff_* = 13.43, CI [1.89, 24.97]) but not when the cue was long (*t*_(6)_ = 2.05, *p* = .09, *M_diff_* = 13.71, CI [-2.70, 30.13]).

4 We also saw priming at 1.5 s in Experiment 3 but somewhat less reliably.
