# Supplementary material for: Exploring the functional nature of synaesthetic colour: Dissociations from colour perception and imagery
Source: Cognition. 2018 Aug;177:107–21. doi: 10.1016/j.cognition.2018.03.022 (PMC6092315; doi:10.1016/j.cognition.2018.03.022)
Supplement: Supplementary data 1 — Supplemental Information includes supplemental analysis, example stimuli, and supplemental details about participation [file mmc1.docx]

Supplemental information **Exploring the nature of synaesthetic experience: dissociations from colour perception and imagery**Rocco Chiou^1,3^, Anina N. Rich^2^, Sebastian Rogers^3^ & Joel Pearson^3^

**^1^** The Neuroscience and Aphasia Research Unit (NARU), Division of Neuroscience and Experimental
 Psychology, University of Manchester, UK

**^2^** Perception in Action Research Centre & Department of Cognitive Science,
 Macquarie University, Sydney, NSW, Australia

**^3^** ARC Centre of Excellence in Cognition and its Disorders, Australia

**^4^** School of Psychology, University of New South Wales, Sydney, NSW, Australia

| **Synaesthete** | **Experiment 1** | **Experiment 2** | **Experiment 3** | **Experiment  4A & 4B** | **Experiment 5** |
| --- | --- | --- | --- | --- | --- |
| CM | *v* | *v* | *v* | *v* | *v* |
| CMM | *v* | *v* |  | *v* | *v* |
| CS |  | *v* |  | *v* |  |
| EB |  | *v* |  | *v* |  |
| FK | *v* |  | *v* |  | *v* |
| HT |  | *v* |  | *v* |  |
| JK | *v* | *v* |  | *v* | *v* |
| JM |  |  | *v* |  |  |
| MY | *v* | *v* | *v* | *v* | *v* |
| RN |  | *v* | *v* | *v* |  |
| RR |  | *v* | *v* | *v* |  |
| SH |  |  | *v* |  |  |
| TD |  | *v* | *v* | *v* |  |
| TS | *v* |  |  |  | *v* |

***Table S1.*** The 14 synaesthetic participants across the 5 experiments. The marks indicate the individuals who participated in each experiment. Note the order pertains to alphabetic sequence, not order of testing *per se*. In the main paper, each synaesthete is denoted by a unique symbol throughout the experiments.

***Categorisation of the 14 synaesthetic participants.***

There is an ongoing debate regarding whether synaesthetes can be reliably classified as ‘associators’ (those who see their synaesthetic colour in the mind’s eye) and ‘projectors’ (those who see it out in space). This classification, originally proposed by Dixon et al. ([Dixon, Smilek, & Merikle, 2004](#_ENREF_3)), has engendered conflicting findings and contention ([for review, see Chiou & Rich, 2014](#_ENREF_2)). Given the controversy, we checked the synaesthetes’ responses on the ‘associator vs. projector’ questionnaire. Consistent with previous observations ([Edquist, Rich, Brinkman, & Mattingley, 2006](#_ENREF_4)), we found that the synaesthetes’ questionnaire responses were not always consistent with a clear categorisation either within a single session or between sessions. Many of the synaesthetes had difficulty describing the subjective locus of their synaesthetic colour percepts – they reported that neither ‘in the mind’s eye’ nor ‘out in space’ fits the spatial frame in which their synaesthetic colour appears exactly. As a result, their answer to the question about locus “*do you experience synaesthetic colour in the mind’s eye or out in space*?” was not always consistent with more specific questions probing the precise location of the experience in the same questionnaire (e.g., “*The colour looks like it is on the page”*). In addition, the same responses were not always given across sessions to the main question of ‘mind’s eye’ vs. ‘out in space’. Despite some positive findings ([Brang, Rouw, Ramachandran, & Coulson, 2011](#_ENREF_1); [Dixon et al., 2004](#_ENREF_3)), there have been numerous studies showing that participants classified as associators and projectors do not reliably differ from each other on various behavioural experiments and their subjective reports tended to vary over time (e.g., [Edquist et al., 2006](#_ENREF_4); [Nijboer, Satris, & Stigchel, 2011](#_ENREF_5); [Nijboer & Van der Stigchel, 2009](#_ENREF_6); [Ward, Jonas, Dienes, & Seth, 2010](#_ENREF_7)), undermining the reliability and validity of this taxonomy. The responses we observed in our sample are consistent with previous results that synaesthetes cannot be reliably categorised under the labels of ‘associators vs. projectors’ using subjective report.

| **Syn.** |  | **Original colour 1** | | | **Colour 1 after dominance test** | | |  | **Original colour 2** | | | **Colour 2 after dominance test** | | |
| --- | --- | --- | --- | --- | --- | --- | --- | --- | --- | --- | --- | --- | --- | --- |
|  |  |  |  |  |  |  |  |  |  |  |  |  |  |  |
| CM |  | 188 | 0 | 0 | 164 | 0 | 0 |  | 0 | 116 | 0 | 0 | 110 | 0 |
| CMM |  | 255 | 5 | 5 | 255 | 5 | 5 |  | 0 | 176 | 80 | 0 | 172 | 78 |
| CS |  | 51 | 51 | 204 | 51 | 51 | 204 |  | 255 | 255 | 0 | 222 | 222 | 0 |
| EB |  | 79 | 98 | 255 | 79 | 98 | 255 |  | 252 | 252 | 86 | 161 | 161 | 55 |
| FK |  | 204 | 0 | 0 | 204 | 0 | 0 |  | 0 | 102 | 77 | 0 | 85 | 64 |
| HT |  | 51 | 51 | 204 | 49 | 49 | 196 |  | 255 | 255 | 0 | 242 | 242 | 0 |
| JK |  | 0 | 132 | 209 | 0 | 132 | 209 |  | 255 | 255 | 0 | 194 | 194 | 0 |
| JM |  | 0 | 0 | 255 | 0 | 0 | 255 |  | 255 | 255 | 0 | 219 | 219 | 0 |
| MY |  | 0 | 49 | 255 | 0 | 49 | 255 |  | 255 | 255 | 0 | 227 | 227 | 0 |
| RN |  | 223 | 11 | 11 | 219 | 11 | 11 |  | 0 | 150 | 0 | 0 | 144 | 0 |
| RR |  | 255 | 0 | 0 | 255 | 0 | 0 |  | 39 | 195 | 31 | 21 | 107 | 17 |
| SH |  | 0 | 0 | 255 | 0 | 0 | 250 |  | 255 | 255 | 87 | 222 | 222 | 76 |
| TD |  | 255 | 0 | 0 | 230 | 0 | 0 |  | 0 | 100 | 0 | 0 | 100 | 0 |
| TS |  | 158 | 18 | 4 | 158 | 18 | 4 |  | 0 | 101 | 0 | 0 | 86 | 0 |

***Table S2. Colours (RGB values) used for each synaesthetes.***

For those who participated in multiple experiments, we used the same RGB triplets of original colours for different experiments. However, the ‘tinkered’ colours after the eye-dominance test might differ slightly between sessions. Here, we provided example values of RGB triplets before (original) and after the eye-dominance test of all of our synaesthetes.


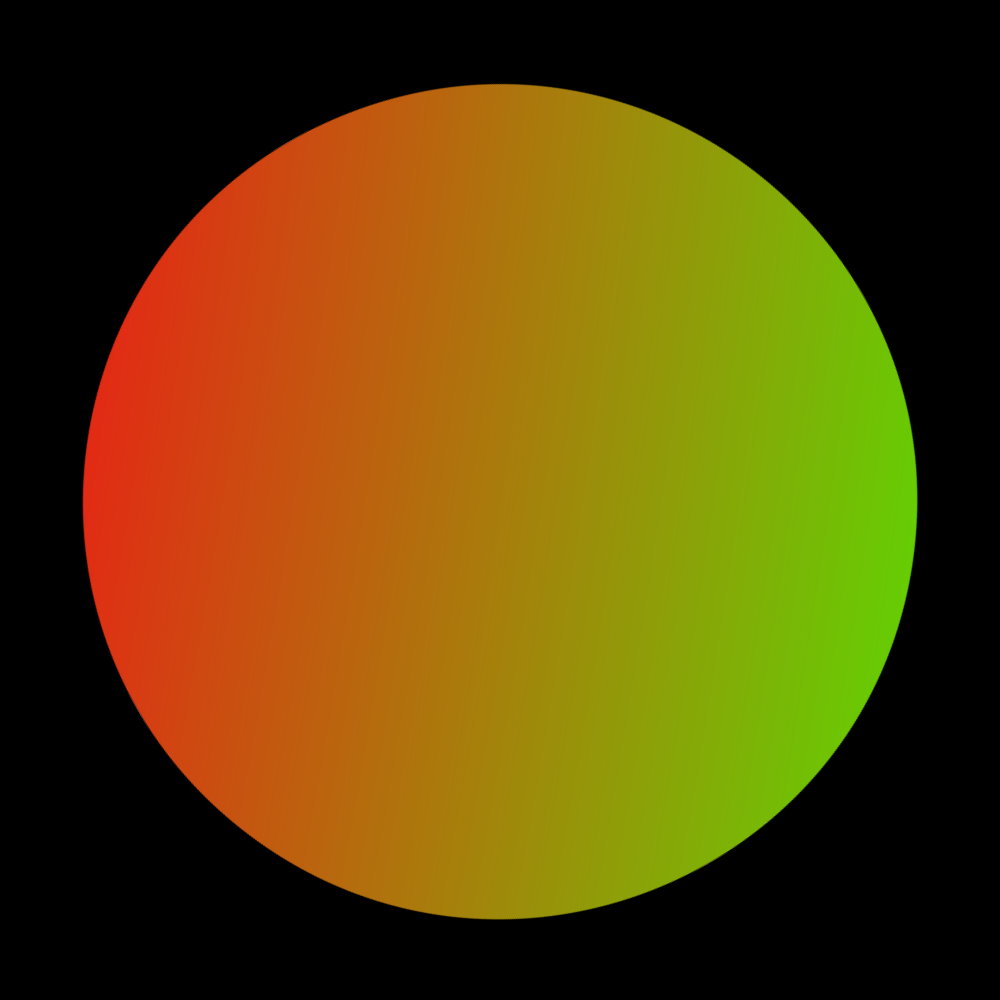

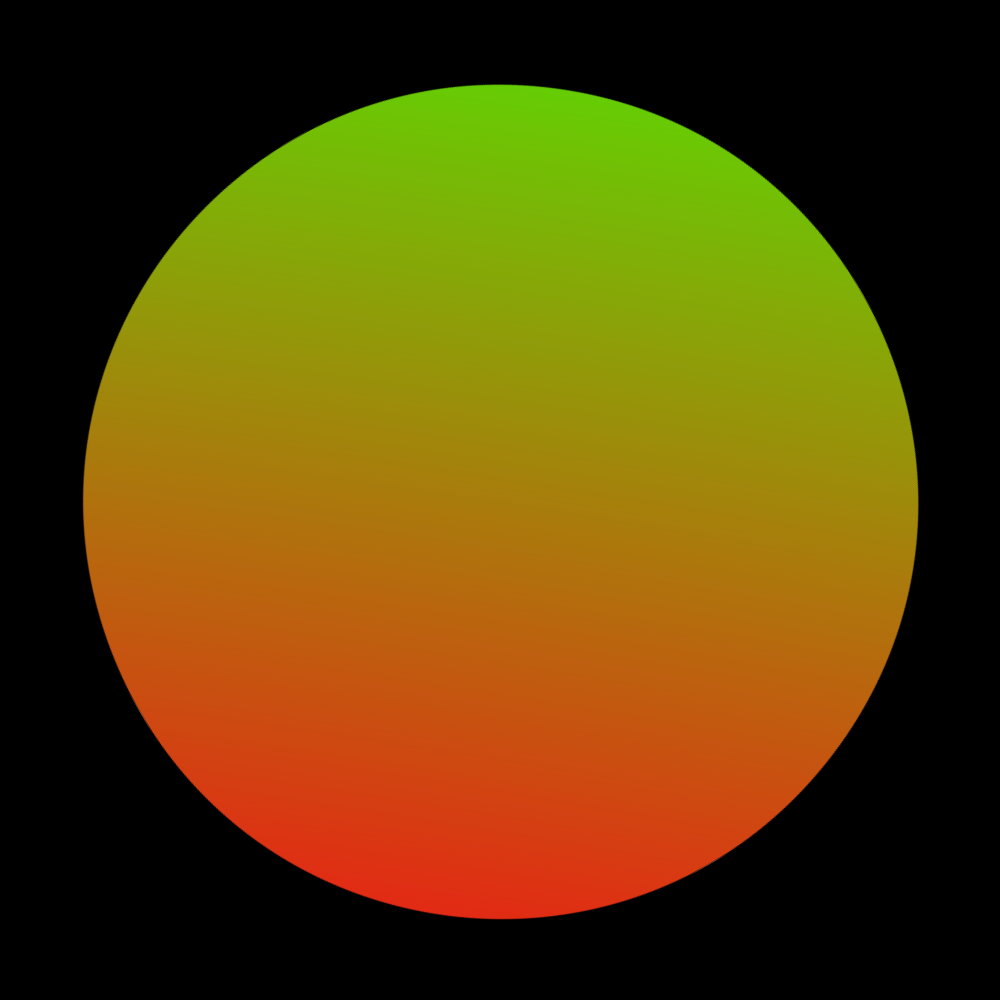


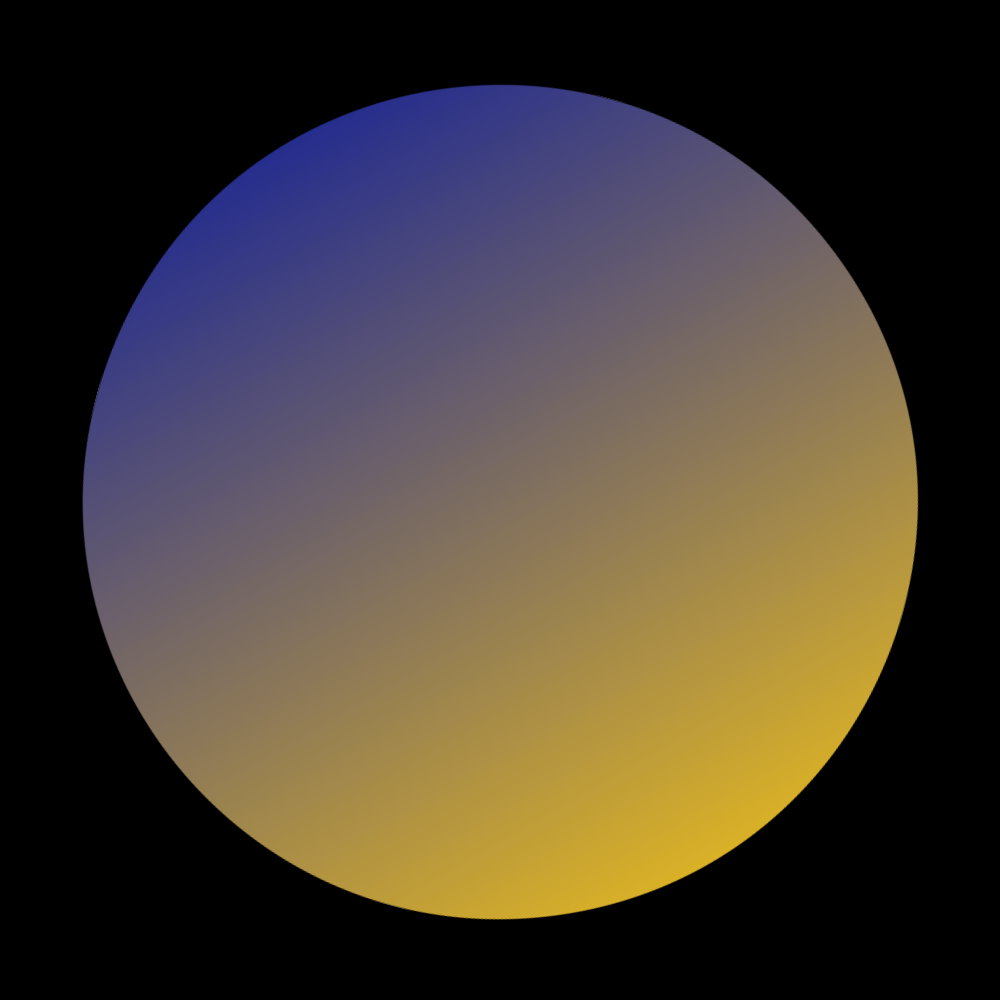

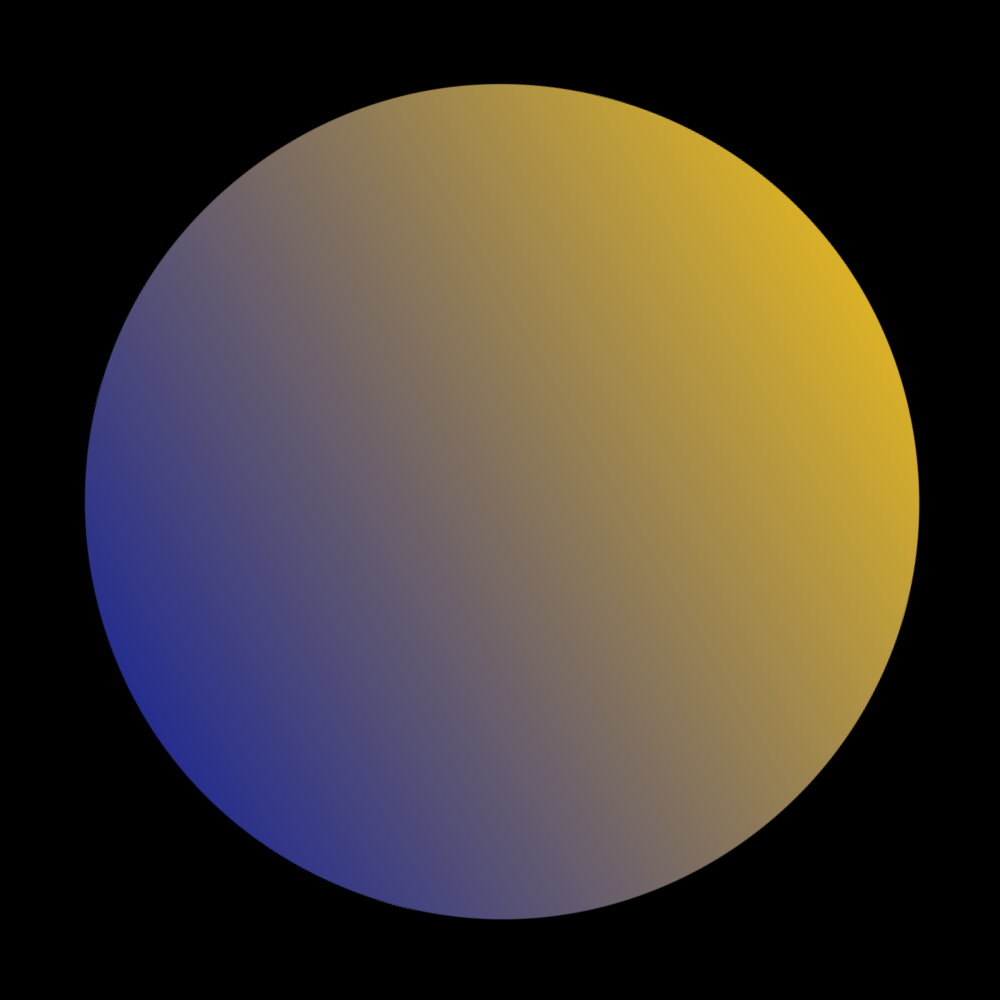


***Figure S1. Examples of mock rivalry stimuli.***

**Supplemental references**

Brang, D., Rouw, R., Ramachandran, V. S., & Coulson, S. (2011). Similarly shaped letters evoke similar colors in grapheme-color synesthesia. *Neuropsychologia, 49*(5), 1355-1358.

Chiou, R., & Rich, A. N. (2014). The role of conceptual knowledge in understanding synaesthesia: Evaluating contemporary findings from a ‘hub-and-spoke’ perspective. *Frontiers in psychology, 5*.

Dixon, M. J., Smilek, D., & Merikle, P. M. (2004). Not all synaesthetes are created equal: projector versus associator synaesthetes. *Cognitive, Affective, & Behavioural Neuroscience, 4*(3), 335-343.

Edquist, J., Rich, A. N., Brinkman, C., & Mattingley, J. B. (2006). Do synaesthetic colours act as unique features in visual search? *Cortex, 42*(2), 222-231.

Nijboer, T. C., Satris, G., & Stigchel, S. V. (2011). The influence of synesthesia on eye movements: No synesthetic pop-out in an oculomotor target selection task. *Consciousness and Cognition, 20*(4), 1193-1200.

Nijboer, T. C., & Van der Stigchel, S. (2009). Is attention essential for inducing synesthetic colors? Evidence from oculomotor distractors. *J Vis, 9*(6), 21 21-29.

Ward, J., Jonas, C., Dienes, Z., & Seth, A. (2010). Grapheme-colour synaesthesia improves detection of embedded shapes, but without pre-attentive 'pop-out' of synaesthetic colour. *Proceedings of the Royal Society: Biological Science, 277*(1684), 1021-1026.
